# Microbial community and soil enzyme activities driving microbial metabolic efficiency patterns in riparian soils of the Three Gorges Reservoir

**DOI:** 10.3389/fmicb.2023.1108025

**Published:** 2023-04-21

**Authors:** Yining Yang, Yao Chen, Zhe Li, Yuanyuan Zhang, Lunhui Lu

**Affiliations:** ^1^Key Laboratory of Hydraulic and Waterway Engineering of the Ministry of Education, Chongqing Jiaotong University, Chongqing, China; ^2^CAS Key Laboratory of Reservoir Water Environment, Chongqing Institute of Green and Intelligent Technology, Chinese Academy of Sciences, Chongqing, China

**Keywords:** riparian soils, microbial metabolic efficiency, soil enzyme activities, microbial community, soil physical and chemical properties

## Abstract

Riparian zones represent important transitional areas between aquatic and terrestrial ecosystems. Microbial metabolic efficiency and soil enzyme activities are important indicators of carbon cycling in the riparian zones. However, how soil properties and microbial communities regulate the microbial metabolic efficiency in these critical zones remains unclear. Thus, microbial taxa, enzyme activities, and metabolic efficiency were conducted in the riparian zones of the Three Gorges Reservoir (TGR). Microbial carbon use efficiency and microbial biomass carbon had a significant increasing trend along the TGR (from upstream to downstream); indicating higher carbon stock in the downstream, microbial metabolic quotient (qCO_2_) showed the opposite trend. Microbial community and co-occurrence network analysis revealed that although bacterial and fungal communities showed significant differences in composition, this phenomenon was not found in the number of major modules. Soil enzyme activities were significant predictors of microbial metabolic efficiency along the different riparian zones of the TGR and were significantly influenced by microbial α-diversity. The bacterial taxa Desulfobacterota, Nitrospirota and the fungal taxa Calcarisporiellomycota, Rozellomycota showed a significant positive correlation with qCO_2_. The shifts in key microbial taxa *unclassified_k_Fungi* in the fungi module #3 are highlighted as essential factors regulating the microbial metabolic efficiency. Structural equation modeling results also revealed that soil enzyme activities had a highly significant negative effect on microbial metabolism efficiency (bacteria, path coefficient = −0.63; fungi, path coefficient = −0.67).This work has an important impact on the prediction of carbon cycling in aquatic-terrestrial ecotones.

**Graphical abstract:**
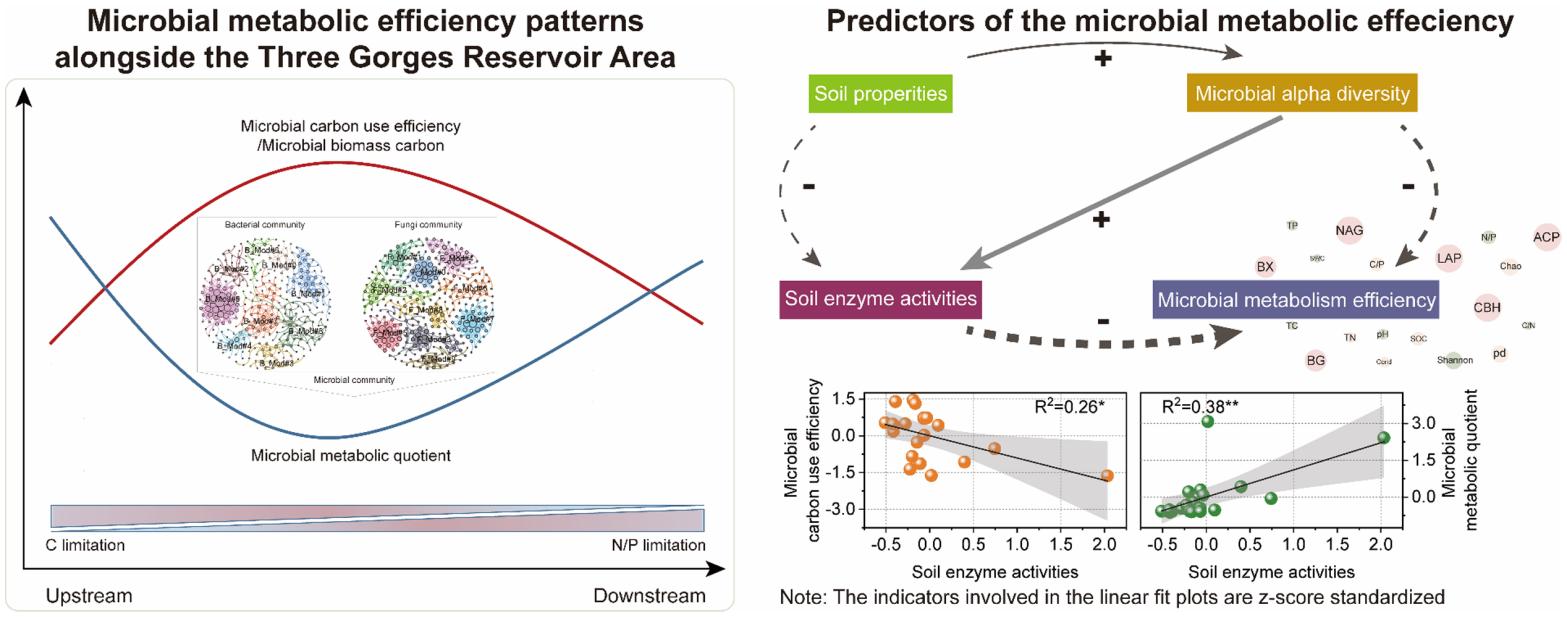

## 1. Introduction

Riparian zones are areas formed along rivers, lakes, and open-water wetlands in the transition area from aquatic to terrestrial ecosystems (Welsh et al., 2017; Hille et al., 2018), and they are water-land interface areas that have both water and land characteristics. The rich biodiversity and unique edge effects make riparian zone habitats dynamic, complex and diverse (Gregory et al., 1991). Riparian soils represent a vital reservoir of biodiversity and underline a multitude of ecosystem processes and functions. Riparian zone soils are an important part of the environmental composition and their biodiversity influences the structural and ecosystem function of the riparian zone.

Microorganisms in riparian soils regulate the main carbon fluxes between the soil and the atmosphere, where they are the key drivers of the carbon cycle. Riparian zones are usually found in channels that are unmanaged and formed by natural water level fluctuations (Malik et al., 2018). Dynamic riparian zone habitats result in the loss of organic carbon in riparian zone soils. Microbial metabolic efficiency, as an important indicator of microbial anabolism, represents the C distribution between microbial biomass and CO_2_ production, and can reflect the changes of microbial physiological characteristics (Frey et al., 2013; Mo et al., 2021). In this study, carbon use efficiency (CUE), microbial metabolic quotient (qCO_2_), microbial biomass turnover time (τ), and microbial biomass carbon (MBC) are defined to evaluate the microbial metabolic effenciency. Generally, lower qCO_2_ and higher CUE indicate higher metabolic efficiency in the soil ecosystems (Wardle and Ghani, 1995; Chen et al., 2018). The CUE is an important regulator of carbon stock, and it can also affect the C retention time and carbon turnover rate of an ecosystem (Wieder et al., 2013; Adingo et al., 2021). It has been shown that microbial communities’ microbial metabolic efficiency such as qCO_2_ and CUE, is the basis of ecosystem carbon storage rates (Xu et al., 2017; Chen et al., 2018; Malik et al., 2018). Some studies have shown that the metabolic efficiency of microbial communities is influenced by abiotic factors and varies with environmental conditions (Sinsabaugh et al., 2013; Xu et al., 2017). Microbial growth and CUE were found to be influenced by microbial diversity and community structure (Soares and Rousk, 2019). Furthermore, soil enzymes are proteins produced by microbial cell secretions, which are involved in the whole process of decomposition and synthesis of organic matter and release of nutrients in the soil (Hill et al., 2012). Riparian soil microbial communities are very sensitive to water-level disturbances and changes in the external environment, and the unique inverse seasonal variation in water level has a great impact on their composition and structural changes, affecting the secretion of soil microbial enzymes, respiratory metabolism and/or catabolism, thus affecting ecological processes closely related to the soil carbon cycle (Allison et al., 2010; Feng et al., 2019).

Microbial community composition and key taxa may also activate soil carbon transformation in various processes. However, despite the recognition that microbial communities are critical for microbial metabolism efficiency, as far as we know, there are still relatively few studies on microbial diversity and microbial metabolic efficiency in riparian soil ecosystems. It is necessary to study the mechanisms by which soil microbial communities regulate their physiological properties (e.g., selective enzyme secretion for nutrient uptake under nutrient-limited conditions; regulation of interspecific community competition or collaboration, etc.) to adapt to external environmental dynamics. Moreover, research has indicated positive relations between biodiversity and soil functions, such as denitrification and methanogenesis (Delgado-Baquerizo et al., 2020). However, changes in microbial communities of soils with rich diversity might be associated with an abundant presence of functionally redundant organisms that generally do not translate into changes in soil function, especially for carbon cycling function (Wertz et al., 2006).

The reservoir riparian zone has a water system that is independent of natural water systems such as streams and rivers. China’s Three Gorges Dam blocks a natural river, creating a large reservoir and a total riparian area of 349 km^2^ (Ye et al., 2019; Zhu et al., 2022). With the implementation of the Three Gorges Dam project in 2008, the reservoir level fluctuates from 145 m in summer (May to September) to 175 m in winter (October to April; Zhang and Lou, 2011). Previous studies have mainly focused on geomorphic delineations (Gurnell et al., 2001; Verry et al., 2004; Clerici et al., 2013), the effects of hydrology (Brosofske et al., 1997; Wantzen et al., 2008), plant colonization (Hupp and Osterkamp, 1996; Li T. et al., 2022), biogeochemical actions (Smith et al., 2012; Zhang et al., 2012), ecological services (Sparovek et al., 2002; Stutter et al., 2012), and interactions among the studied objects (Gregory et al., 1991; Osterkamp and Hupp, 2010; Polvi et al., 2011; Gurnell et al., 2012; Ding et al., 2022). However, the impacts of dams on hydrologic and biogeochemical processes in the riparian zones of reservoirs could be more complex and diverse. The unique inverse seasonal variation in water level has a great impact on changes in soil conditions and vegetation types (Ye et al., 2012; Garssen et al., 2015), and these changes ultimately affect soil composition and soil enzyme activity as well as soil functions. Sensitive riparian habitats might establish complex interaction characteristics between microorganisms and microbial metabolism efficiency. It is undoubtedly important to study links between microbial communities and soil carbon functionality, which can provide valuable information on microbial predictions of ecosystem processes and functions in the riparian soils.

The overall object of this study is to explore the direct or indirect drivers on microbial metabolic efficiency along the riparian zones of the TGR. We hypothesize that: (1) The sensitive and complex riparian habitats results different microbial metabolic efficiency distribution in the riparian zones of the TGR; (2) More diverse in soil microbial communities will have higher microbial metabolic efficiency; and (3) High soil enzyme activities means higher microbial capacity to utilize substrates, thus soil enzyme activities might can be indicators of microbial metabolic efficiency in the riparian ecosystems. To overcome these key issues, we select the riparian soils in the TGR to conduct relative researches. Our work shows that the impact of the microbial communities, diversity, and soil enzyme activities on microbial metabolic efficiency.

## 2. Materials and methods

### 2.1. Experimental design and sampling campaigns

Sampling campaigns were conducted at the Three Gorges Reservoir (TGR), China. This reservoir is approximately 662.9 km, spanning from Chongqing (west) to Yichang, Hubei (east; Chang et al., 2010). The water level is impounded to 175 m for power generation in the winter and discharged to 145 m for flood control in the summer, forming a unique artificial riparian zone that totals approximately 349 km^2^ (Yang et al., 2012; Zhu et al., 2022).

**Figure 1:**
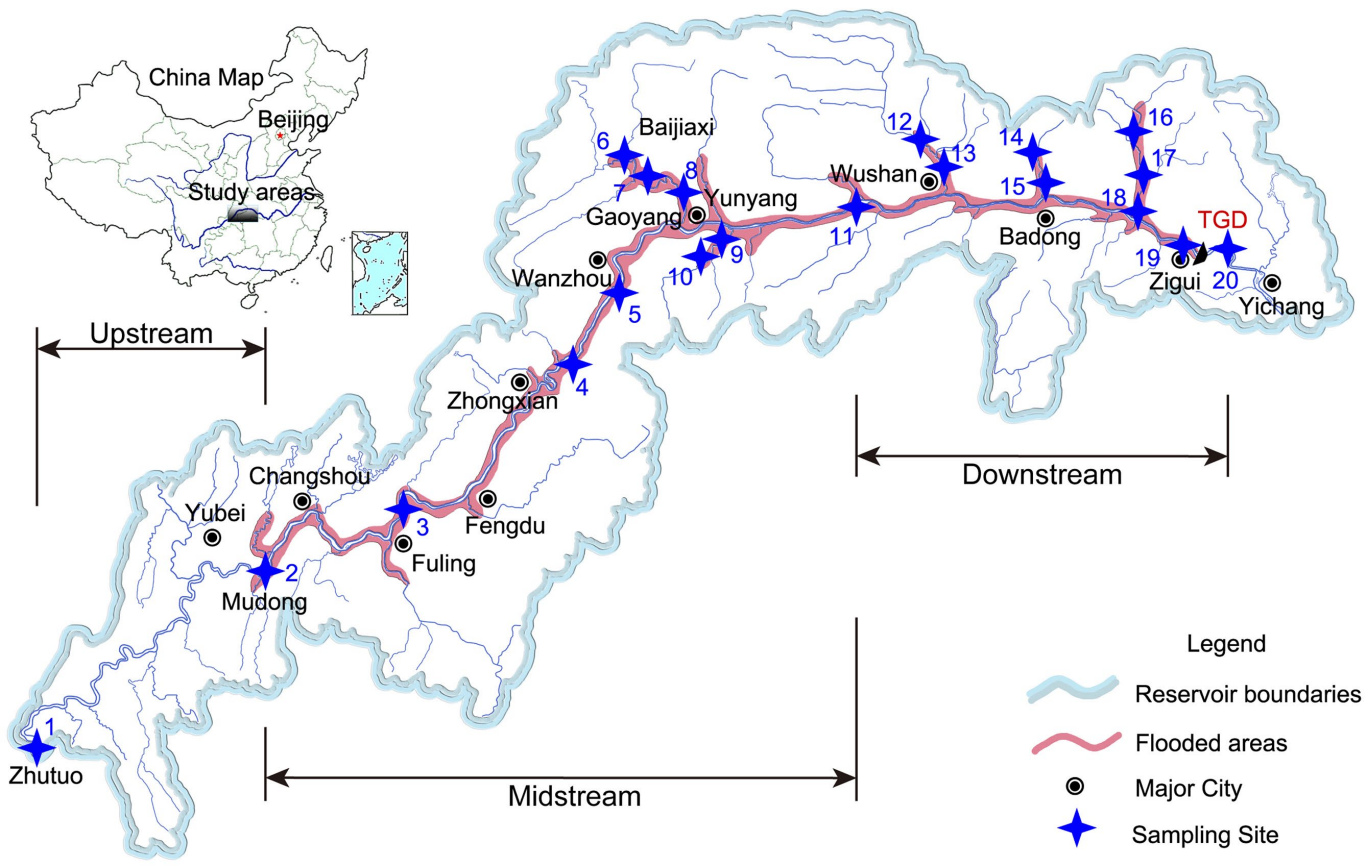
Location of sampling sites in the riparian zone of Three Gorges Reservoir, China.

The field sampling campaigns were carried out in December 2021. A total of 20 sampling sites were selected in the riparian zone along the TGR (Figure 1), including two sites upstream, eight sites midstream, and 10 sites downstream. Detailed geographic information of the sampling sites is given in Supplementary Table S1. Total 4–5 different portions of riparian zone topsoil (0–20 cm) were collected within 1 m^2^ using a shovel, and the soil was thoroughly mixed and reduced to 1 kg by quadratic fractionation after removing obvious impurities such as plant roots and stones. Samples were then encapsulated in polyethylene self-sealing bags and placed in low-temperature, sterile containers (so that the internal temperature was maintained between 2 and 6°C) and sent immediately to the laboratory. On arrival at the laboratory, the samples were divided into three parts: those for microbial community characterization were immediately frozen at −80°C, those for soil water content (SWC), MBC, and soil extracellular enzyme activities were stored at 4°C, and those for physicochemical analyses were air-dried and then sieved before use.

### 2.2. Soil properties

The pH of riparian soil was determined by the 1:2.5 (w/v) electrode method. Samples were dried at 105°C for 24 h to determine SWC. Soil total carbon (TC) and total nitrogen (TN) were measured by an elemental analyzer (Vario PYRO Cube, Elementar, Germany). Soil total phosphorus (TP) was analyzed according to the Standards, Measurements, and Testing (SMT) methods (Ruban et al., 2001; Sun et al., 2021; Appendix S1). Three parallel samples were provided for quality control.

### 2.3. Microbial metabolic efficiency

In this study, microbial metabolic efficiency was defined as an important indicator of microbial anabolism, evaluated with CUE, MBC, τ, and qCO_2_. Since microorganisms have a preference for different carbon substrates, measuring CUE by labeling the substrate carbon would bias the results (Schwartz, 2007). Based on the results of previous studies, it was shown that more than 90% of the oxygen in the process of DNA synthesis by microbial growth comes from water-oxygen, and the results are reliable (Schwartz, 2007; Li et al., 2016; Spohn et al., 2016a). Therefore, we measured CUE using a substrate carbon-independent ^18^O-H_2_O chamber culture method, and simultaneously measured microbial basal respiration and τ (Spohn et al., 2016a; Qu et al., 2020). The CUE was measured as follows: We weighed 6 g of fresh soil in a 150 mL plastic wide-mouth bottle and added a certain amount of ultrapure water to adjust the water content to 60% of the field Water Holding Capacity (WHC), and place it in a pre-culture at 20°C for 24 h. Afterward, six pre-cultured soil samples (0.5 g each) were taken into 50 mL culture flasks with screw caps, three of which were spiked with 100 μL of ^18^O-H_2_O (20.0 atom%^18^O, Campro Scientific, Germany) for labeling experiments and the other with an equal amount of ultrapure water as a natural abundance control. Seal an empty vial at three sample intervals to obtain a control sample of laboratory air at the start of the incubation. The vials were incubated in a constant temperature incubator at 20°C for 48 h. After incubation, 15 mL of gas was extracted from each vial using a syringe with a Luer lock and transferred to an evacuated vacuum bag (0.3 L, HEDE tech, Dalian, China), and timely measurement of CO_2_ concentration by gas chromatograph (Agilent 8860 GC System, Spanish). Soil respiration rate was quantified as μgCO_2_-C g^−1^ dry soil h^−1^. After the gas samples were taken, the culture flasks were removed and placed in a freeze dryer for the freeze-drying process until DNA extraction. Total soil DNA was extracted using a DNA extraction kit according to the manufacturer’s procedures.^1^ DNA concentrations were then quantified by Picogreen fluorescence analysis (Quant-iT™ PicoGreen® dsDNA Reagent, Thermo Fisher, Germany) using a microplate spectrophotometer (Infinite® M200, Tecan, Austria). The remaining DNA extracts were then transferred to silver cups, and placed in an oven at 45°C until dry, then the packaged samples were sealed and the ^18^O isotope abundance and O content were determined using a stable isotope mass spectrometer (Thermo Fisher Scientific, MA, United States). Based on the steady-state assumption, the amount of carbon absorbed by microbial biomass (C_Uptake_) is calculated as follows.


(1) $$C_{\mathrm{Uptake}}=C_{\mathrm{Growth}}+C_{\mathrm{Respiration}}$$

Where C_Growth_ is the carbon flux allocated to biomass production (growth) and C_Respiration_ is the carbon flux allocated to CO_2_ production (respiration).

Microbial CUE is then calculated by the following equation (Manzoni et al., 2012; Sinsabaugh et al., 2013).


(2) $$\mathrm{CUE}=\frac{C_{Growth}}{C_{Uptake}}$$

Microbial biomass carbon was determined by the chloroform-fumigation extraction method (Vance et al., 1987; Setia et al., 2012), details of the experimental procedure and the calculation of MBC are given in the Supplementary material (Appendix S2). qCO_2_ was expressed as μg CO_2_-C (μg MBC)^−1^ h^−1^ (Wardle and Ghani, 1995), and was calculated by the ratio of C_Respiration_ to MBC, referring to the calculation in previous studies (Zheng et al., 2019). τ was calculated by the ratio of MBC to C_growth_ with reference to previous research methods (Spohn et al., 2016a).

### 2.4. Prediction of soil enzyme activities and nutrient limitation in riparian soil ecosystems

Soil enzyme activities related to carbon [β-1,4-glucosidase (BG), β-xylosidase (BX), cellobiose hydrolase (CBH), and polyphenol oxidase (PPO)], nitrogen [N-acetyl-β-D-glucosaminidase (NAG) and leucine aminopeptidase (LAP)], and phosphorus [acid phosphatase (ACP)] were determined according to the enzyme activity assay kit.^2^ All enzyme activities were measured by a fluorometric method in 96-well microplates using a multimode microplate reader (Infiniti M200PRO, Switzerland; Marx et al., 2001; Wang et al., 2021). Extracellular enzyme activities were expressed as nmol h^−1^ g^−1^ soil.

Furthermore, enzyme activities were normalized by MBC to avoid the variations induced by biomass change. In this study, the enzyme stoichiometric vector model was used to calculate microbial metabolic restriction characteristics (Moorhead et al., 2013, 2016).

Where Length and Angle are, respectively, calculated by equations (3) and (4).


(3) $$\mathrm{Length}=\sqrt{x^{2}+y^{2}}$$

(4) Angle=degrees(atan2(x,y))

Where x = (BG + CBH)/(BG + CBH + ACP) and y = (BG + CBH)/(BG + CBH + LAP+NAG). A higher Length value indicates relatively higher C vs. nutrient acquisition strategies, and a higher Angle value suggests higher P vs. N acquisition efforts.

Here, the soil enzyme activity index was calculated based on the average of all single enzyme activities measured (Luo et al., 2018), and was used as a general index that could reflect the change in the extracellular enzyme activity of the soil microorganism. Before quantifying this index, all single enzyme activity indices were normalized by Z-scores (Wagg et al., 2014).

### 2.5. Microbial communities and bioinformatics analysis

Following the manufacturer’s instructions, soil DNA was extracted by using the Powersoil® DNA Isolation Kit (MoBio, CA, United States). Subsequently, primer pairs 338F/806R (Huws et al., 2007) and ITS1F/ITS2R (White, 1990) were used to amplify bacterial 16S rRNA and fungal ITS coding genes. Afterward, the purified amplicons were pooled in equimolar amounts and paired-end sequenced on an Illumina MiSeq platform at Majorbio Bio-Pharm Technology Co., Ltd., Shanghai, China.

All bioinformatics analyses were based on amplicon sequence variants (ASVs; Callahan et al., 2017), using DATA2 denoising to remove any low-quality reads, and then clustering the eligible merged sequences into ASVs (Callahan et al., 2016). In this study, alpha diversity indices [Chao1, Shannon, and phylogenetic diversity (PD)] were calculated according to the 97% ASV similarity of the sequences. Modules are highly connected regions in a network that may reflect the aggregation of phylogenetically closely related species, overlapping niches and the co-evolution of species, and they are considered phylogenetically, evolutionarily, or functionally independent units (Olesen et al., 2007). ASVs with high Spearman correlation coefficients (|*r*| > 0.8) and statistically significant (*p* < 0.05) correlations were selected for bacterial and fungal contribution network analysis to identify the major eco-clusters (modules or assemblages) of strongly correlated ASVs (Li H. et al., 2022). The network core node discrimination methods of within-module connectivity (*Z*_i_) and among-module connectivity (*P*_i_) have been widely applied, based on this, we used them for inference of network node properties and filtering of key species (Deng et al., 2012). Further bioinformatics analysis is available in the Supplementary material (Appendix S3).

### 2.6. Other data analysis and statistical tests

The distribution of microbial metabolism-related indicators along the TGR was evaluated using OriginPro 2022 (OriginLab Corporation, MA, United States) in a violin plot. Statistical differences in microbial alpha diversity upstream and downstream were tested by one-way ANOVA. Spearman’s rank correlation analysis was used to assess the relationship between soil microbial carbon metabolism and soil physical–chemical properties and microbial extracellular enzyme activities. It was also used to evaluate the relationship between keystone taxa in the microbial network and key modules in the microbial community. A random forest analysis was performed to determine statistically significant predictors of microbial metabolism efficiency (CUE, MBC, qCO_2_, and τ) using the *rePermute* package (Breiman, 2004) in R (version 4.1.3).

The direct and indirect effects of soil physical–chemical properties, microbial alpha diversity, and soil enzyme activities on microbial metabolic efficiency were evaluated by a structural equation model (SEM). The hypothesized path structure was based on the proposition that abiotic drivers can drive microbial metabolic efficiency not only directly, but also indirectly drive it by influencing the biotic factors (Supplementary Figure S1). We infer that: (1) Soil physical and chemical properties can directly affect microbial α diversity, soil enzyme activities, and microbial metabolic efficiency; (2) Microbial α diversity drives soil enzyme activities and microbial metabolic efficiency, and the influence of bacterial and fungal α diversity on them is different; and (3) Soil enzyme activities have direct effect on microbial metabolic efficiency. Due to the strong Spearman correlation between the factors in each group, before constructing the SEM, principal component (PC) analysis was first performed to establish multivariate functional relationships, thereby integrating multiple single variables into one composite variable (Chen et al., 2019). The first component (PC1) explained 70.64–91.34% of the total variance of those four groups, and PC1 was then brought in as a composite variable to the subsequent analysis species to express the group properties of the combination (Supplementary Table S2). Finally, the goodness of fit of the SEM was checked by the χ^2^ test and the root mean square error of approximation (Chen et al., 2019). Analysis of the structural equation model was performed using AMOS 26.0 (AMOS Development Corporation, Chicago, IL, United States).

## 3. Results

### 3.1. Microbial metabolism indicators and resource acquisition traits in riparian soils

**Figure 2:**
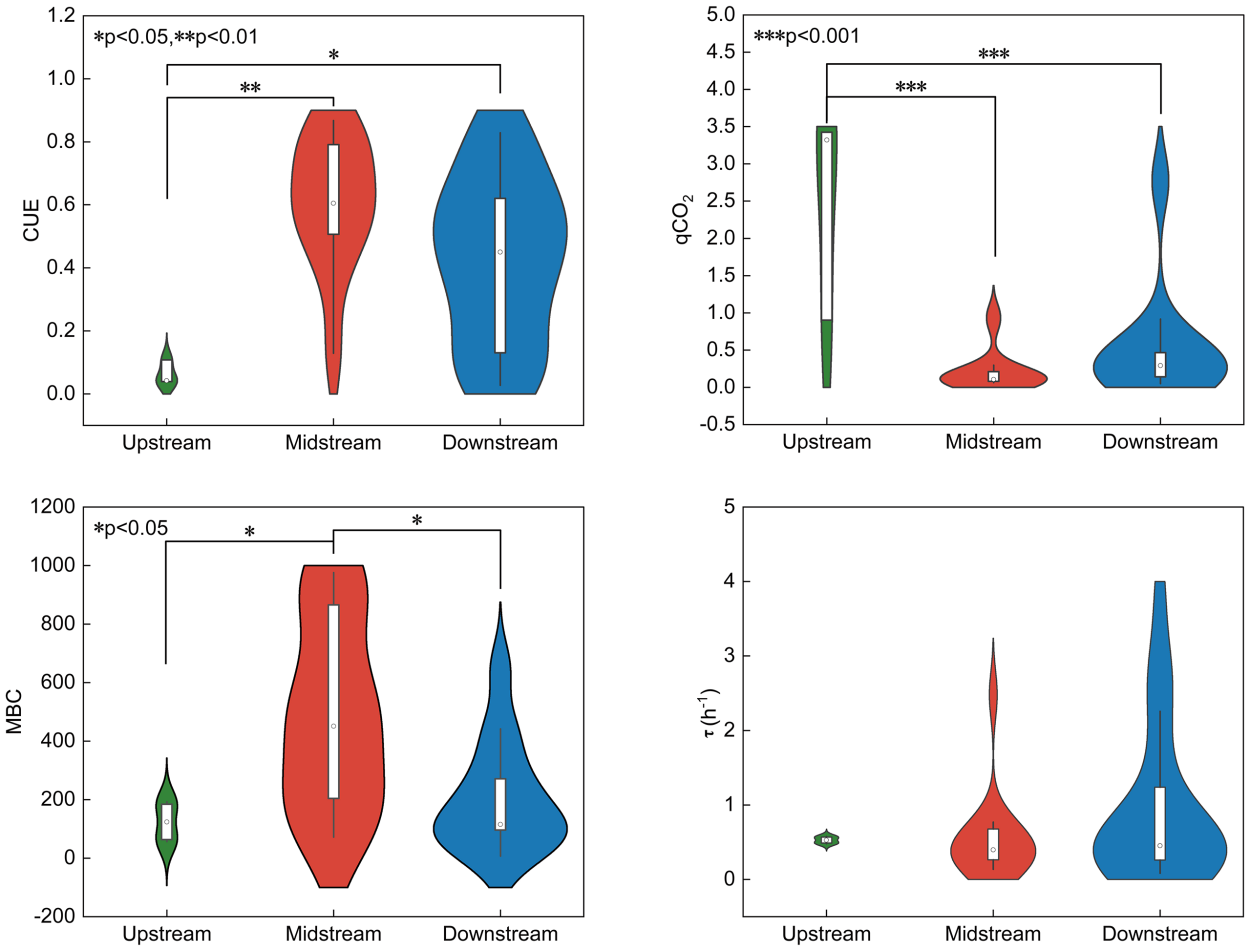
Soil carbon metabolism indicators along the different riparian zones (upstream, midstream and downstream) of TGR. CUE, carbon use efficiency; MBC, microbial biomass carbon; qCO_2_, metabolic quotient; τ, microbial biomass turnover time. ^*^p < 0.05, ^**^p < 0.01, ^***^p < 0.001.

The microbial metabolism indicators (including CUE, qCO_2_, MBC, and τ) along the TGR were showed in Figure 2. Microbial CUE and MBC had a significant increasing trend along the TGR (from the upstream to downstream), and the qCO_2_ showed an opposite trend, indicating there are higher metabolic efficiency in the downstream of the TGR. There was no significant change in τ (Figure 2). According to the enzyme metric vector model (Supplementary Figure S2), the soil microbial communities were mostly limited by soil phosphorus and partially limited by soil nitrogen along the TGR riparian zone.

### 3.2. Soil microbial diversity and communities in riparian zones

**Figure 3:**
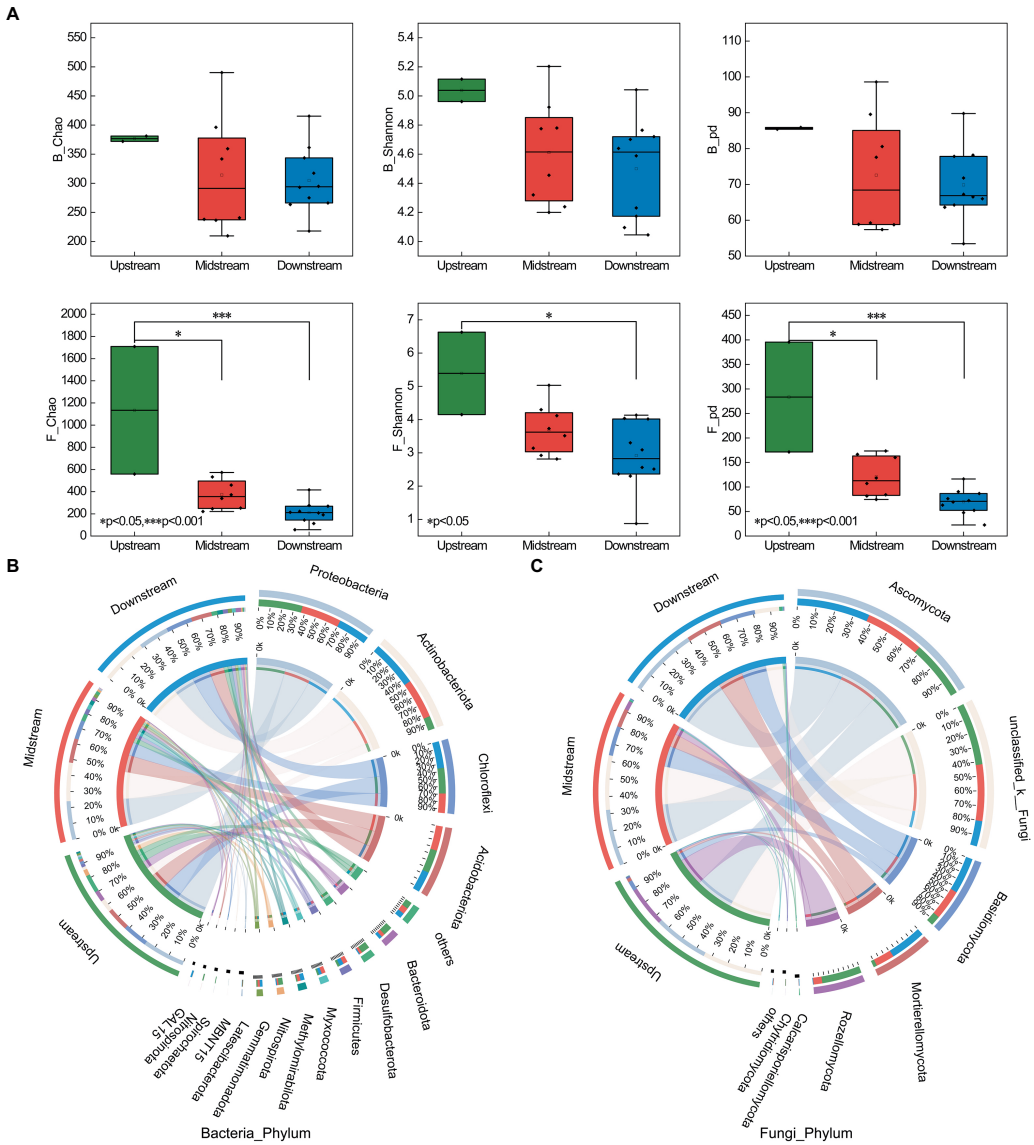
The alpha diversity and microbial communities in riparian zones of the TGR. **(A)** Distribution of alpha diversity (bacteria and fungi) in the riparian zone (upstream, midstream, and downstream). **(B)** Relative abundance of bacterial communities at the phylum level (upstream, midstream, and downstream); B_Chao, Chao index of bacterial community; F_Chao, Chao index of fungal community. **(C)** Relative abundance of fungal communities at the phylum level (upstream, midstream, and downstream). ^*^p < 0.05, ^**^p < 0.01, ^***^p < 0.001.

Overall, the bacterial and fungal alpha diversity (including Chao, Shannon, and PD indices) showed a decreasing trend from upstream to downstream. The fungal alpha diversity showed highly significant differences between upstream and downstream (Chao and PD indices; Figure 3). A total of 15,269 ASVs and 5,469 ASVs were detected for the bacterial and fungal communities, respectively. In the TGR, the Shannon diversity of bacterial communities was higher than that of fungal communities (Supplementary Figure S3).

The abundant bacterial phyla at the community phylum level were Actinobacteriota (25.41%), Proteobacteria (21.65%), Chloroflexi (15.35%), and Acidobacteriota (14.07%; Supplementary Figure S6). The abundant fungal phyla at the community phylum level were Ascomycota (36.6%), unclassified_k_Fungi (23.8%), Basidiomycota (17.7%), and Mortierellomycota (14.8%; Supplementary Figure S6). The proportion of microbial community composition also differed significantly along the different areas of the riparian zone (Supplementary Table S3; Supplementary Figure S3).

### 3.3. Co-occurrence network analysis

**Figure 4:**
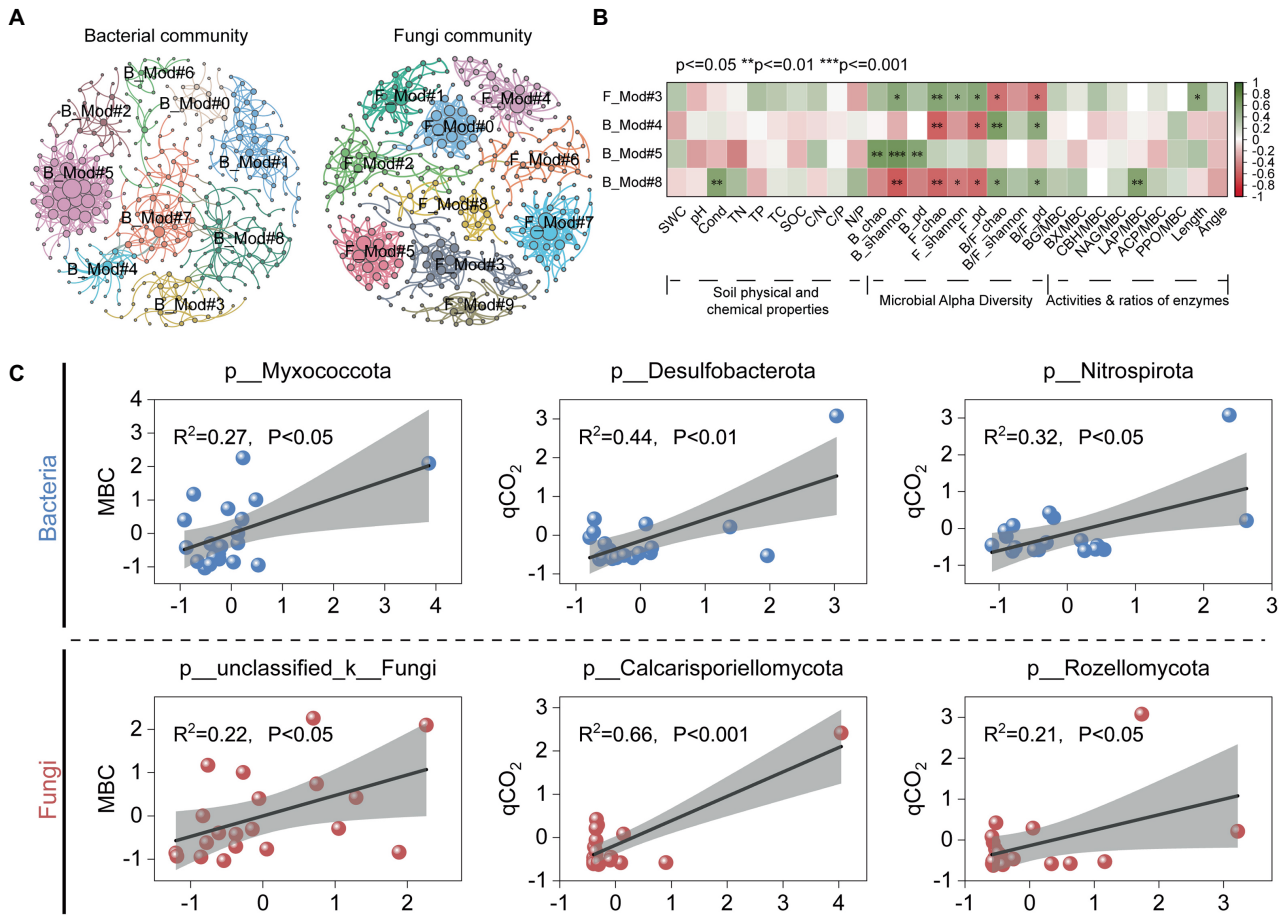
Division of main bacterial and fungal modules and correlation analysis with each index. **(A)** Diagram of network module division, where modules are divided by different colors, the left is bacterial community and the right is fungal community; **(B)** Spearman analysis between network modules and factors (physical–chemical properties, alpha diversity, and enzyme activities). **(C)** Linear fit of dominant species (including bacterial and fungal phylum levels) to microbial metabolism efficiency. ^*^p < 0.05, ^**^p < 0.01, ^***^p < 0.001.

**Figure 5:**
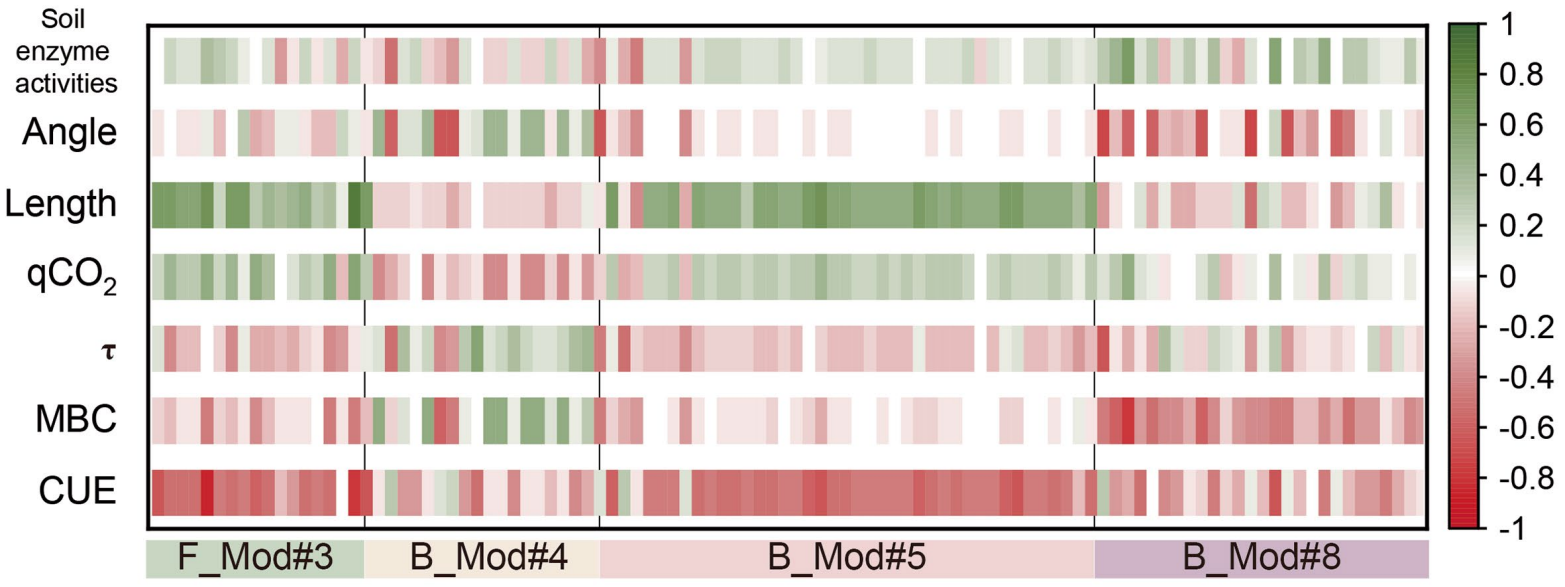
Relationships of the keystone genera with metabolic efficiency, microbial physiological traits and soil enzyme activities. F_Mod#3, key fungal assemblies in module#3; B_Mod#4, B_Mod#5, and B_Mod#8 were key bacterial assemblies in module#4, module#5, and module#8. Length, the relative C: nutrient-acquiring traits.

Microbial co-occurrence networks can generally be divided into multiple modules. Soil bacterial and fungal co-occurrence networks were classified into 9 (B_Mod#0–8) and 10 (F_Mod#0–9) major microbial modules, respectively (Figure 4A). Among them, the relative abundance of phylogenetic types belonging to F_Mod#3 was positively correlated with B_Shannon, F_Shannon and B_pd, F_pd. B_Mod#5 consisted mainly of Proteobacteria, Actinobacteriota, and Desulfobacterota (Supplementary Table S8), and the relative abundance of phylogenetic types of this module was positively correlated with bacterial alpha diversity (B_Chao, B_Shannon, and B_pd). In addition, there were significant positive correlations between the relative abundance of B_Mod#8 and soil conductivity (Cond) as well as soil LAP enzyme activity (Figure 4B). By establishing correlation analysis of key assemblies in the microbial network modules with soil enzyme activities and microbial metabolic efficiency, it was found that most taxa in the network modules were positively correlated with qCO_2_ and nutrient acquisition length (Figure 5; Supplementary Table S8). Chloroflexi in B_Mod#8 showed a relatively strong positive correlation with soil enzyme activities (Supplementary Tables S8, S9). Most taxa in F_Mod#3 showed a significantly positive association with qCO_2_ and Length, but a negative association with CUE (Figure 5).

Moreover, correlations between dominant phylum and microbial metabolism effiency were analyzed (Figure 4C; Supplementary Table S5). It was found that bacterial phylum Myxococcota and the fungal phylum unclassified_k_Fungi were positively correlated with MBC significantly, and the bacterial phylum Desulfobacterota, Nitrospirota and the fungal phylum Calcarisporiellomota, Rozellomota had a significantly positive correlation with qCO_2_ (Figure 4C; Supplementary Table S5). Interestingly, at the key genus level, none of the key genera were significantly correlated with microbial metabolic efficiency, except for the fungal genus *unclassified_k_Fungi*, which showed a significant positive correlation with MBC (Supplementary Table S4).

### 3.4. Linking biotic and abiotic factors to microbial metabolic efficiency

**Figure 6:**
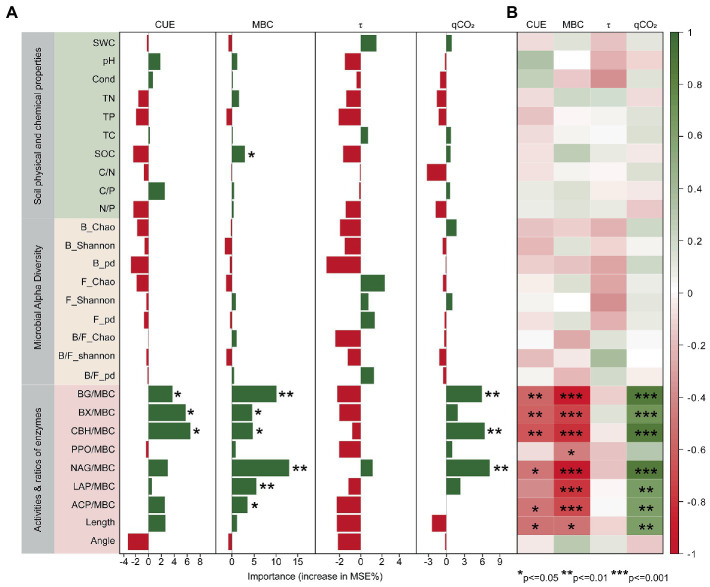
Major predictors of microbial metabolic efficiency. **(A)** Based on the percentage increase in mean squared error (%IncMSE) from the random forest analysis. **(B)** Spearman correlation analysis of microbial carbon metabolism indicators with selected biotic and abiotic factors. Enzyme/MBC is calculated by normalizing the activity to units/mg MBC and represents the specific enzyme activity. ^*^p < 0.05, ^**^p < 0.01, ^***^p < 0.001.

Biotic and abiotic factors were linked to indicators of microbial metabolic efficiency by using random forest and correlation analysis (Figure 6). The results showed that soil enzyme activities were significant predictors of CUE, MBC and qCO_2_ (Figure 6A). Spearman’s correlation (Figure 6B) and redundancy analysis (Supplementary Figure S7B) further indicated that both CUE and MBC showed negative correlations with soil enzyme activities, while qCO_2_ was highly significantly and positively correlated with them (except for PPO enzyme activity). Furthermore, among the soil abiotic factors, SOC was an important predictor of MBC (Figure 6A; Supplementary Figure S7A).

**Figure 7:**
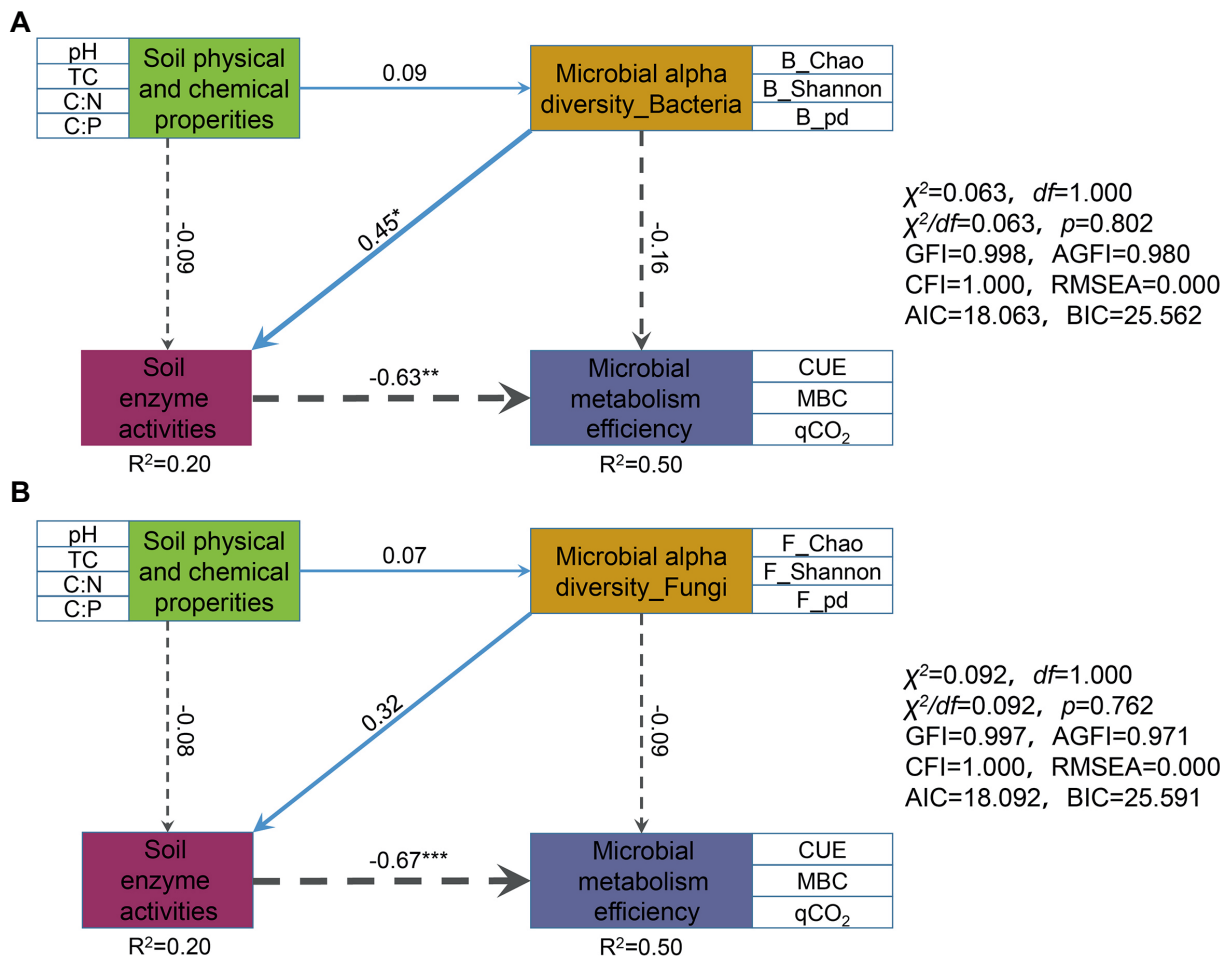
Effects of soil physicochemical properties, microbial alpha diversity, and soil enzyme activities on microbial metabolic efficiency directly and indirectly. The structural equation models (SEM) was constructed for bacteria and fungi respectively: **(A)** microbial alpha diversity_Bacteria; **(B)** microbial alpha diversity_Fungi. Blue solid and gray dotted arrows, respectively, represent positive and negative relationships. The wider the width of the arrow indicates the stronger the correlation. Numbers on arrows are standardized path coefficients. *R*^2^ indicates the proportion of variance explained by predictors. ^*^p < 0.05, ^**^p < 0.01, ^***^p < 0.001. The soil physical and chemical properties, microbial alpha diversity, and microbial metabolism efficiency were represented by the first component of the PCA performed in a multilayer rectangle.

In addition, we use structural equation modeling (SEM) to test whether the relationship between them. Overall, the SEM results also revealed that soil enzyme activities had a highly significant negative effect on microbial metabolism efficiency (bacteria, path coefficient = −0.63; fungi, path coefficient = −0.67; Figure 7). Microbial alpha diversity had a weak effect on microbial metabolic efficiency (bacteria, path coefficient = −0.16; fungi, path coefficient = −0.09), mainly by through affecting enzyme activities and thus indirectly affecting microbial metabolic efficiency (Figure 7).

## 4. Discussion

This study selected 20 representative sampling sites along the TGR to elucidate the change patterns of microbial α-diversity and microbial metabolic efficiency in the riparian soils, and analyzed the driving factors (key taxa, micribial diversity, enzyme activities, and physico-chemcal factors) of the metabolic efficiency patterns using random forest and SEM.

### 4.1. Microbial metabolic efficiency patterns along the riparian zones of the TGR

With the regular impoundment and discharge of water, the TGR has formed a unique riparian zone with a large area. A riparian zone is an ecological area between aquatic and terrestrial regions, with multiple ecosystem functions, such as biodiversity conservation (Mander et al., 2005), riparian stabilization and non-point sources of pollution interception (Salemi et al., 2012). Current research in the riparian zone of the TGR has focused on the effects of hydrological status on the nutrient dynamics of riparian vegetation in the reservoir area (Chen et al., 2021, 2022), the effects of dry and wet cycles or water level fluctuations on soil aggregates and the response of soil microbial communities to external disturbances (e.g., elevated nitrogen levels, different land use types, hydrological stress, etc.; Nsabimana et al., 2020; Ding et al., 2021; He et al., 2021; Li et al., 2021; Nsabimana et al., 2021).

This study provides the first preliminary exploration of microbial metabolic efficiency alongside the riparian zones of the TGR in the high water-level operation period. It was interesting that soils in the upstream showed the lowest CUE, the highest qCO_2_, and diversity of soil microorganisms (Figures 2, 3A). This result may be due to the higher nutrient availability in the upstream region, resulting in higher bacterial community alpha diversity (Yang et al., 2019). Theoretically, soil nutrient limitation controls microbial metabolic processes, including influencing microbial metabolic rates and resource use efficiency. In this work, the lowest CUE and highest qCO_2_ in the upstream is that microorganisms are mainly influenced by carbon limitation (Supplementary Figure S2). There are some publications also showing that the higher carbon limitation results lower CUE in soil ecosystems, additionally, their results showed that qCO_2_ depended not only on the soil carbon concentration but also on the soil C:N and C:P mol ratios (Spohn and Chodak, 2015; Cui et al., 2021). Soils mainly showed phosphorus-limited characteristics in the midstream riparian zone (Supplementary Figure S2), and phosphorus limitation might probably suppress the soil priming effect (Spohn et al., 2016b), thus resulting a relatively higher CUE.

### 4.2. Key taxa in bacterial and fungal assemblies driving the microbial metabolic efficiency

Based on patterns of co-occurrence networks, microbial communities can be classified as assemblies with specific combinations of characteristics, providing new insights into the structure and function of complex microbial communities (Ma et al., 2016). Our study established a relationship between the key taxa in the microbial assemblies and metabolic efficiency. F_Mod#3 showed relatively significant positive and negative correlations with qCO_2_ and CUE, respectively (Figure 5). This is probably because the microbial taxa in F_Mod#3 have slow growth rates (Feng et al., 2021). Among them, the F_Mod#3 module is mainly composed of the dominant phylum Ascomycota (77.78%; Supplementary Table S8), which is mainly saprophytic and parasitic. Ascomycota plays a crucial role in the degradation of various organic substances such as cellulose, cellulose disaccharides and lignin, and the intensity of activity may depend on the expression of the cellobiose dehydrogenase gene (Harreither et al., 2011). It is noteworthy that the genera *Aspergillus* showed a significantly negative correlation with CUE (Supplementary Table S8). The distribution of Ascomycetes in the topsoil of arid ecosystems has been confirmed (Porras-Alfaro et al., 2017;Challacombe et al., 2019; Zhao et al., 2019). They have an important function in soil stability, plant biomass decomposition and are the main functional group for carbon degradation (Challacombe et al., 2019; Zhao et al., 2019). Among them, *Aspergillus* has a strong potential function for lignin degradation (mainly phenol oxidase genes) during the succession of biological soil crusts (Zhao et al., 2019). However, the direct association between Ascomycota and CUE has not been determined to date, which may require further analysis (Fierer and Jackson, 2006).

Key species in microbial communities, community interactions, and community assembly processes are significant predictors of microbial metabolism efficiency (Cui et al., 2018; Zheng et al., 2018). Microorganisms of the K-strategy grow slower but are more efficient in resource utilization, usually have a higher CUE and tend to live in nutrient-deficient environments, and many studies consider fungi to be in this category (Soares and Rousk, 2019; Zhong et al., 2020). In contrast, microorganisms with r-strategies are more metabolically efficient, have higher nutrient requirements, and have lower CUE, such as bacteria (Soares and Rousk, 2019; Zhong et al., 2020). Myxococcota showed a significant positive correlation with MBC (Figure 4C). Desulfobacterota, Nitrospirota, Calcarisporiellomycota, and Rozellomycota were all observed to be significantly and positively correlated with qCO_2_ in this study (Figure 4C). Members of Myxococcota are rare bacterial predators with a unique “wolf-pack hunting” strategy (Petters et al., 2021). It has been confirmed that their metabolism is active *in situ* in the soil microbial food web (Lueders et al., 2006). Desulfobacterota (formerly Deltaproteobacteria) are mainly mesophilic anaerobes, and members of the class Desulfobacterota are best known for their respiration of sulfate (Waite et al., 2020). Members of Nitrospirota (formerly Nitrospirae or Nitrospira) can oxidize nitrite to nitrate and play an important role in denitrification (Ehrich et al., 2007). They are mainly involved in N cycling processes in the soil, thus indirectly influencing the respiratory metabolic capacity of microorganisms, which partially explains the increase in qCO_2_ with the enhanced activity of Desulfobacterota and Nitrospirota, while they did not show a significant association with CUE, MBC (Figure 4C).

### 4.3. Multiple drivers on microbial metabolism efficiency

The relative importance of soil physical–chemical properties, microbial α-diversity, and soil enzyme activities on microbial metabolic efficiency was discerned by constructing an SEM. Here, bacterial α-diversity was found to show a significant positive effect on soil enzyme activities (Figure 7A), which is consistent with the results of the linear relationship of soil enzyme activities (Supplementary Figure S8), and the effect of bacterial α-diversity on soil enzyme activity and microbial metabolic efficiency was stronger than that of fungi (path coefficients of 0.45 > 0.32, |−0.16| > |−0.09|, respectively).

Enzyme activity has a crucial role in the study carbon cycle. Although our study showed a weak and non-significant negative correlation between microbial alpha diversity and microbial metabolism efficiency (bacteria, path coefficient = −0.16; fungi, path coefficient = −0.09. Figure 7), the ratio of bacterial/fungal alpha diversity (e.g., B/F_Chao, B/F_ Shannon, and B/F_pd) was significantly influenced by soil enzyme activity (Supplementary Figure S10). Based on the SEM and RDA, we speculate that microbial alpha diversity can indirectly have a major impact on the metabolic efficiency of microorganisms by significantly influencing enzyme activities (Figure 7; Supplementary Figure S9). In this study, the SEM results were consistent with the random forest results. Microbial enzyme activity was stressed as an important predictor of microbial metabolic efficiency based on the results of random forest results (Figure 6A). The potential extracellular enzyme activity was significantly negatively with CUE and MBC (Figure 6B), supporting the idea of previous studies that the enzyme pool represents a cost hindering growth efficiency (Manzoni et al., 2012; Sinsabaugh et al., 2013; Malik et al., 2019). Prior studies also confirmed that β-glucosidases and ligninases play an essential role in the microbial involvement of soil carbon cycling (Lladó et al., 2017).

## 5. Conclusion

In summary, as far as we know, this study is the first preliminary exploration of the links among microbial metabolic efficiency, microbial alpha diversity and soil enzyme activities in the riparian zone ecosystem. Microbial alpha diversity showed a strong positive correlation with soil enzyme activities, while soil enzyme activities showed a highly significant negative correlation with microbial metabolic efficiency. Our results demonstrate the crucial role of soil enzyme activities in predicting microbial metabolism efficiency. There may be important implications of this work for changes in the carbon cycling of riparian zone ecosystems in the TGR.

## Data availability statement

The datasets presented in this study can be found in online repositories. The names of the repository/repositories and accession number(s) can be found below: https://www.ncbi.nlm.nih.gov/, PRJNA900697 and https://www.ncbi.nlm.nih.gov/, PRJNA900703.

## Author contributions

YY: methodology, data analysis, and writing–original draft. YC: review and editing. ZL: review and editing and funding acquisition. YZ: investigation and review and editing. LL: investigation, experiment, conceptualization, writing–original draft, review and editing, and funding acquisition. All authors contributed to the article and approved the submitted version.

## Funding

The National Natural Science Foundation of China (Project 42107273) and Key Project of the Ministry of Water Resources (SKS-2022081) primarily supported this study. The “Light of West” Program from the Chinese Academy of Sciences provides partial research funds for ZL and LL for this study.

## Conflict of interest

The authors declare that the research was conducted in the absence of any commercial or financial relationships that could be construed as a potential conflict of interest.

## Publisher’s note

All claims expressed in this article are solely those of the authors and do not necessarily represent those of their affiliated organizations, or those of the publisher, the editors and the reviewers. Any product that may be evaluated in this article, or claim that may be made by its manufacturer, is not guaranteed or endorsed by the publisher.

## Footnotes

^1^ https://www.tiangen.com/

^2^ https://www.boxbio.cn/
