# Supplementary figures and images for: Microbial community and soil enzyme activities driving microbial metabolic efficiency patterns in riparian soils of the Three Gorges Reservoir
Source: Front Microbiol. 2023 Apr 21;14:1108025. doi: 10.3389/fmicb.2023.1108025 (PMC10171112; doi:10.3389/fmicb.2023.1108025)

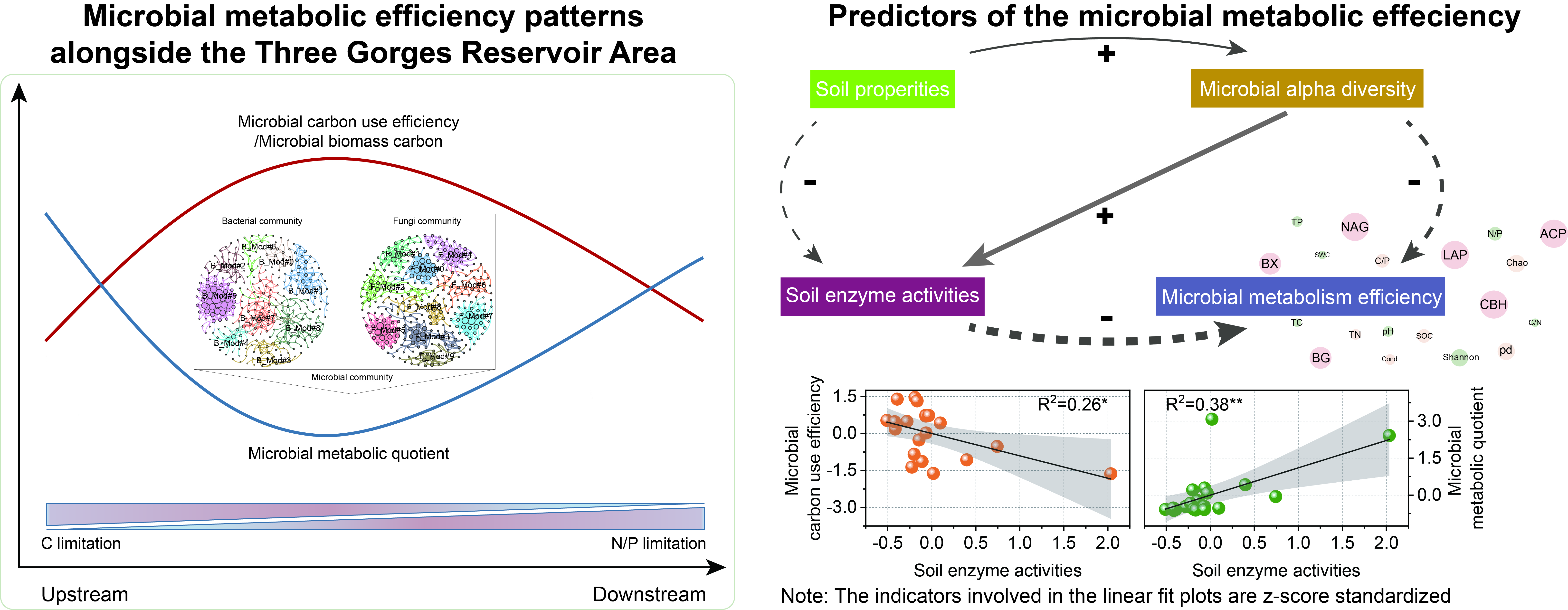

Supplement: Supplementary file 1 [file Image_1.JPEG]

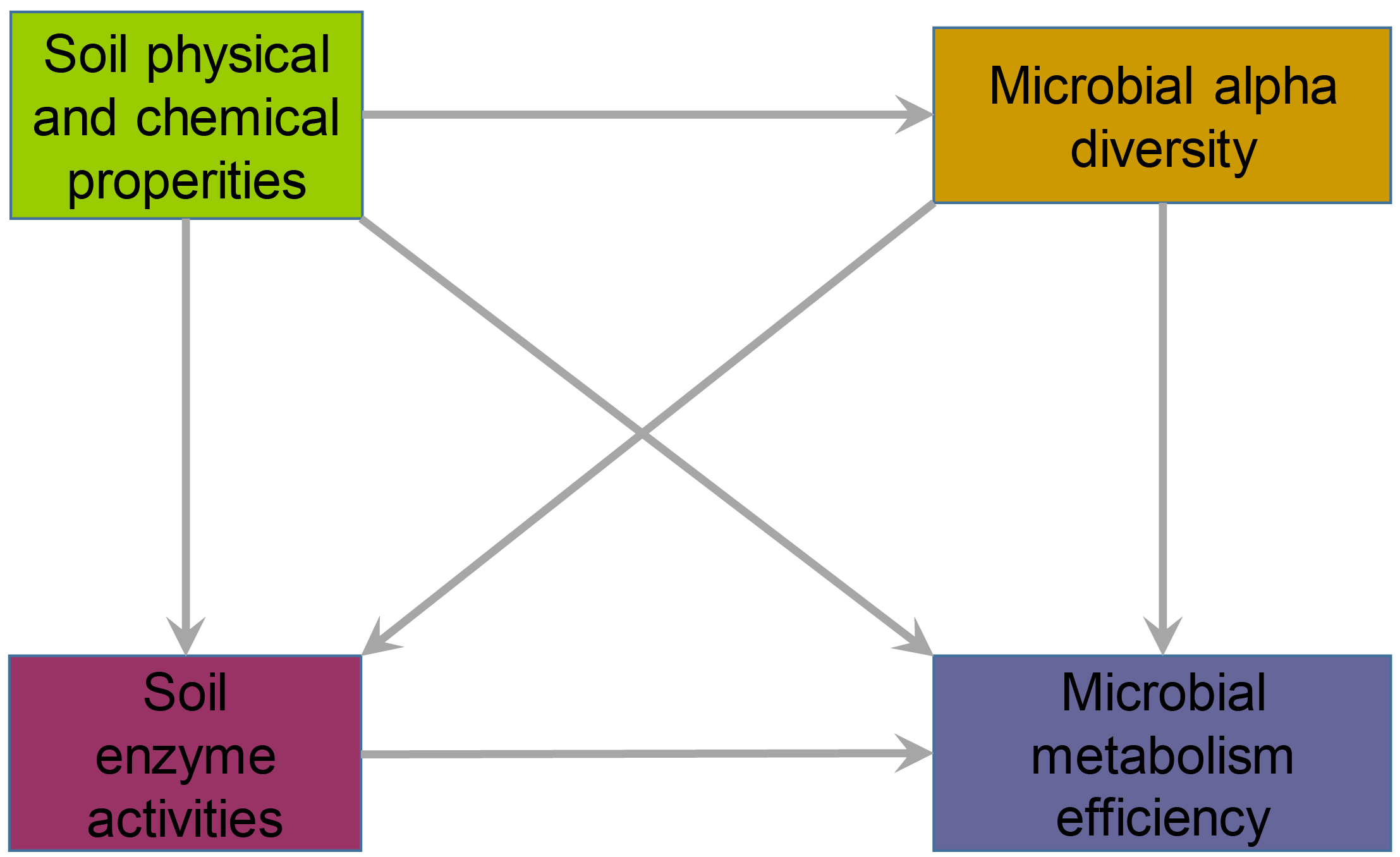

Supplement: Supplementary file 2 [file Image_2.jpg]

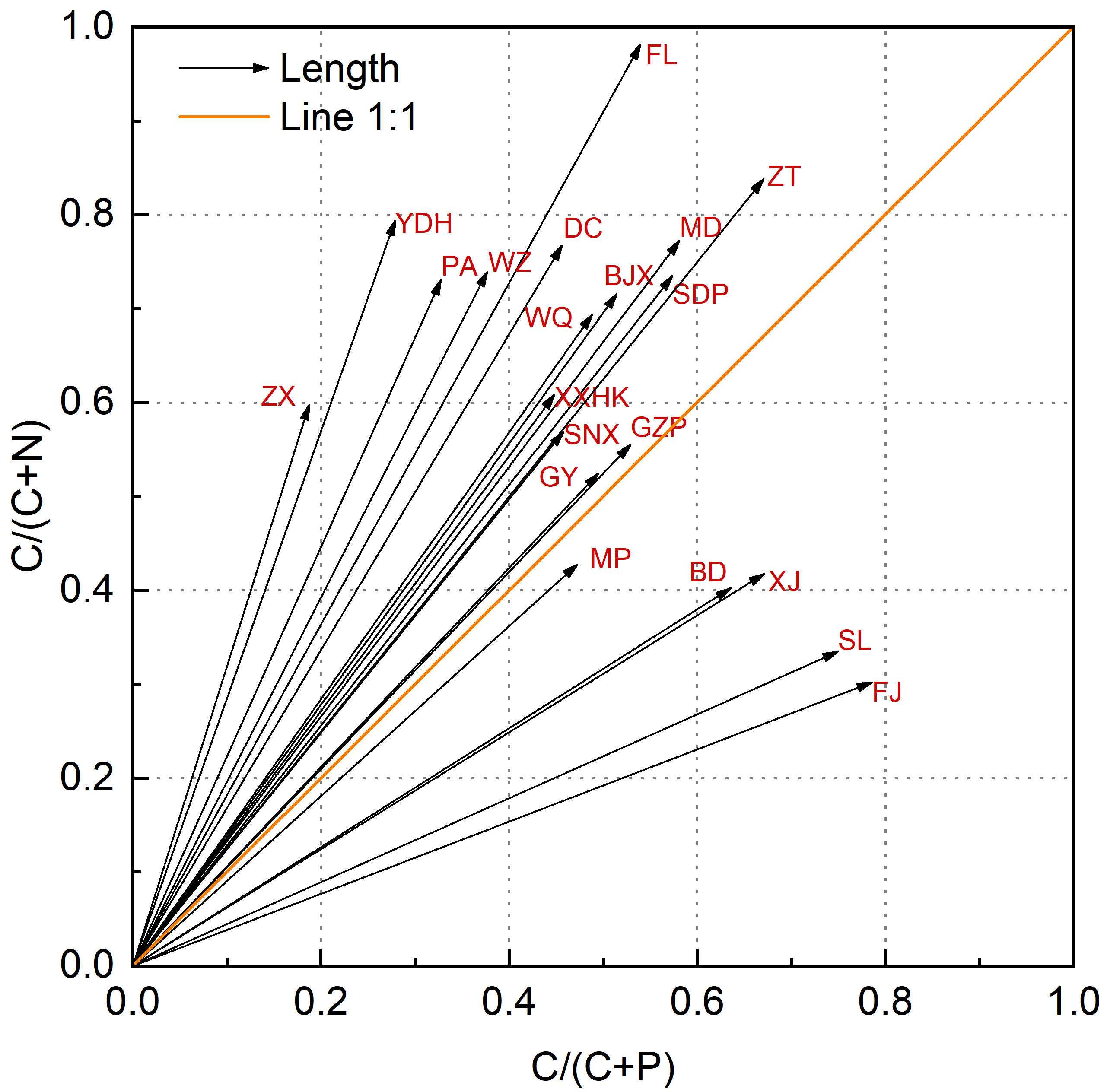

Supplement: Supplementary file 3 [file Image_3.JPEG]

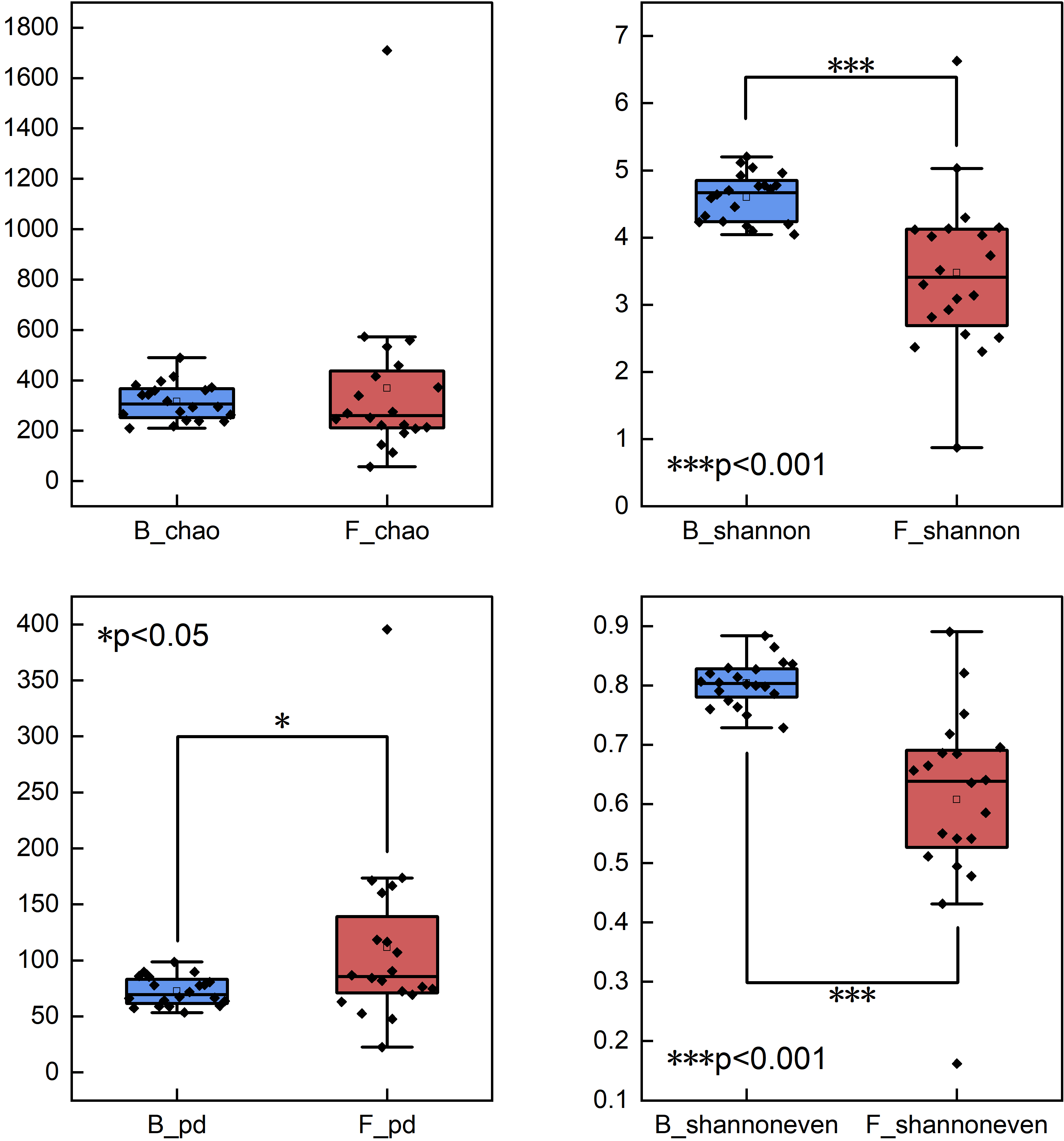

Supplement: Supplementary file 4 [file Image_4.JPEG]

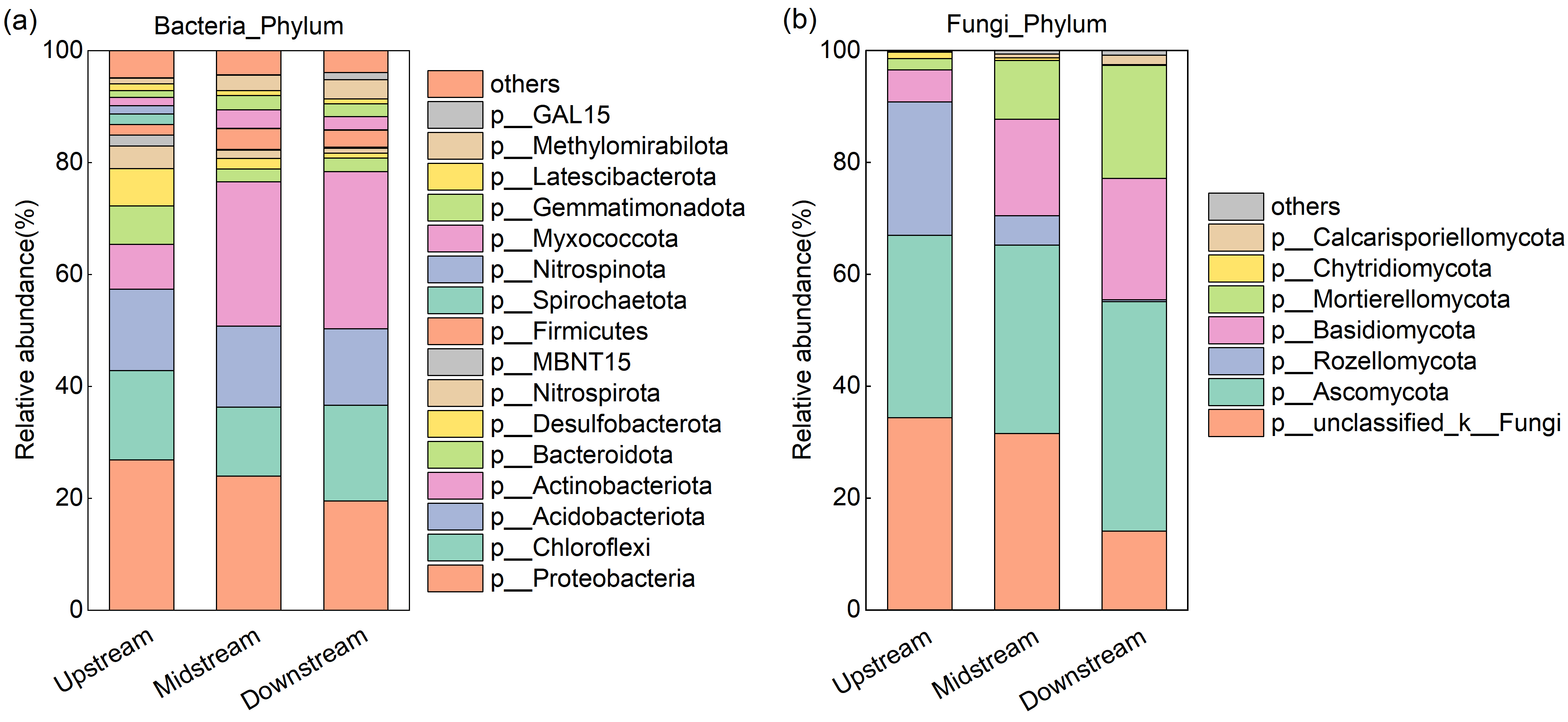

Supplement: Supplementary file 5 [file Image_5.JPEG]

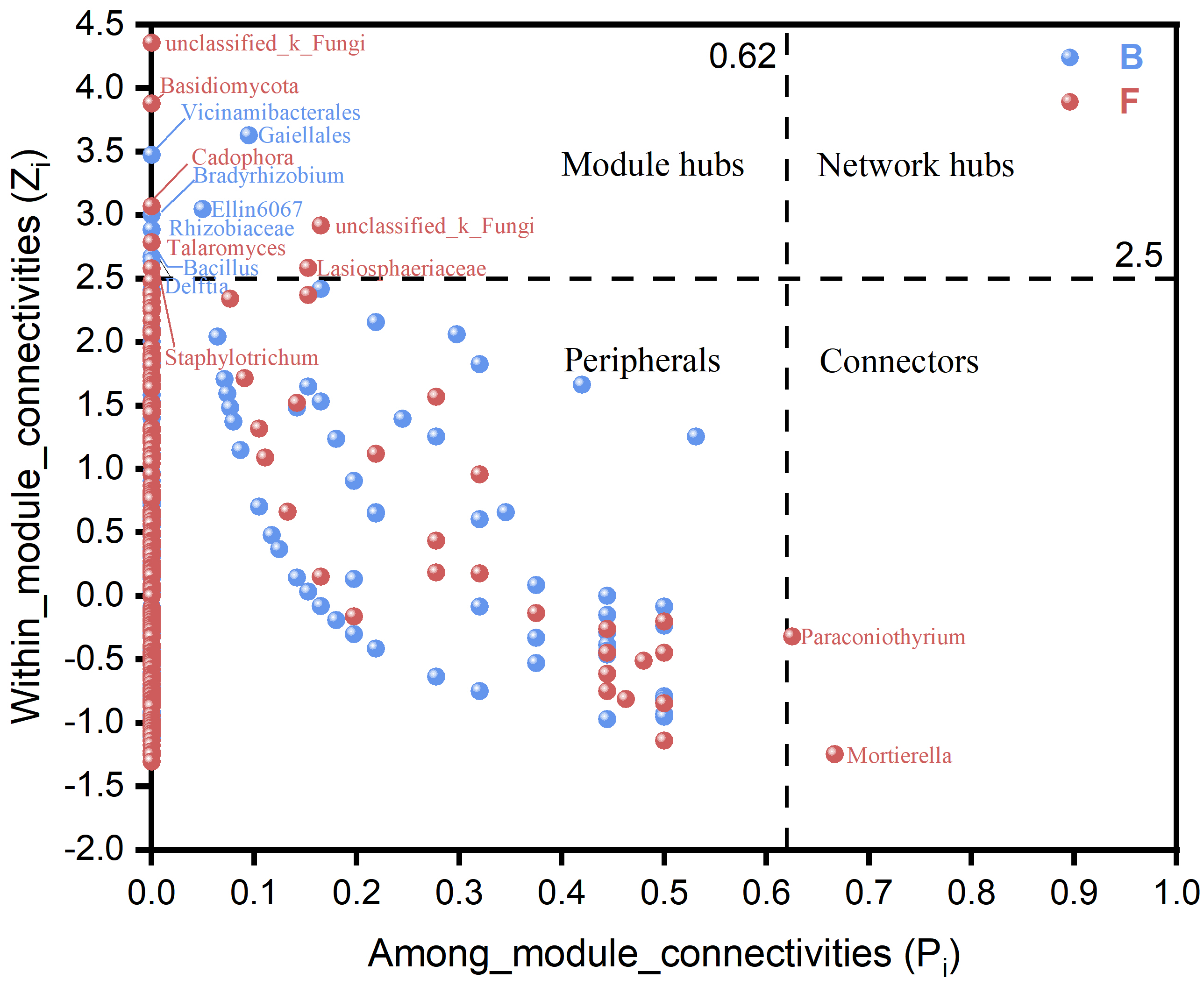

Supplement: Supplementary file 6 [file Image_6.JPEG]

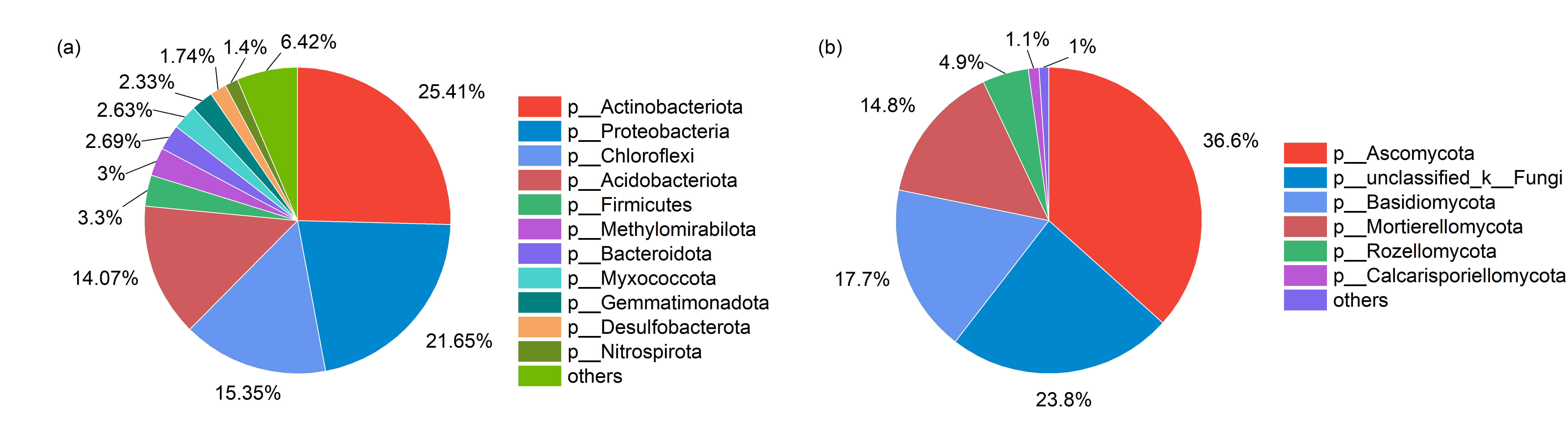

Supplement: Supplementary file 7 [file Image_7.JPEG]

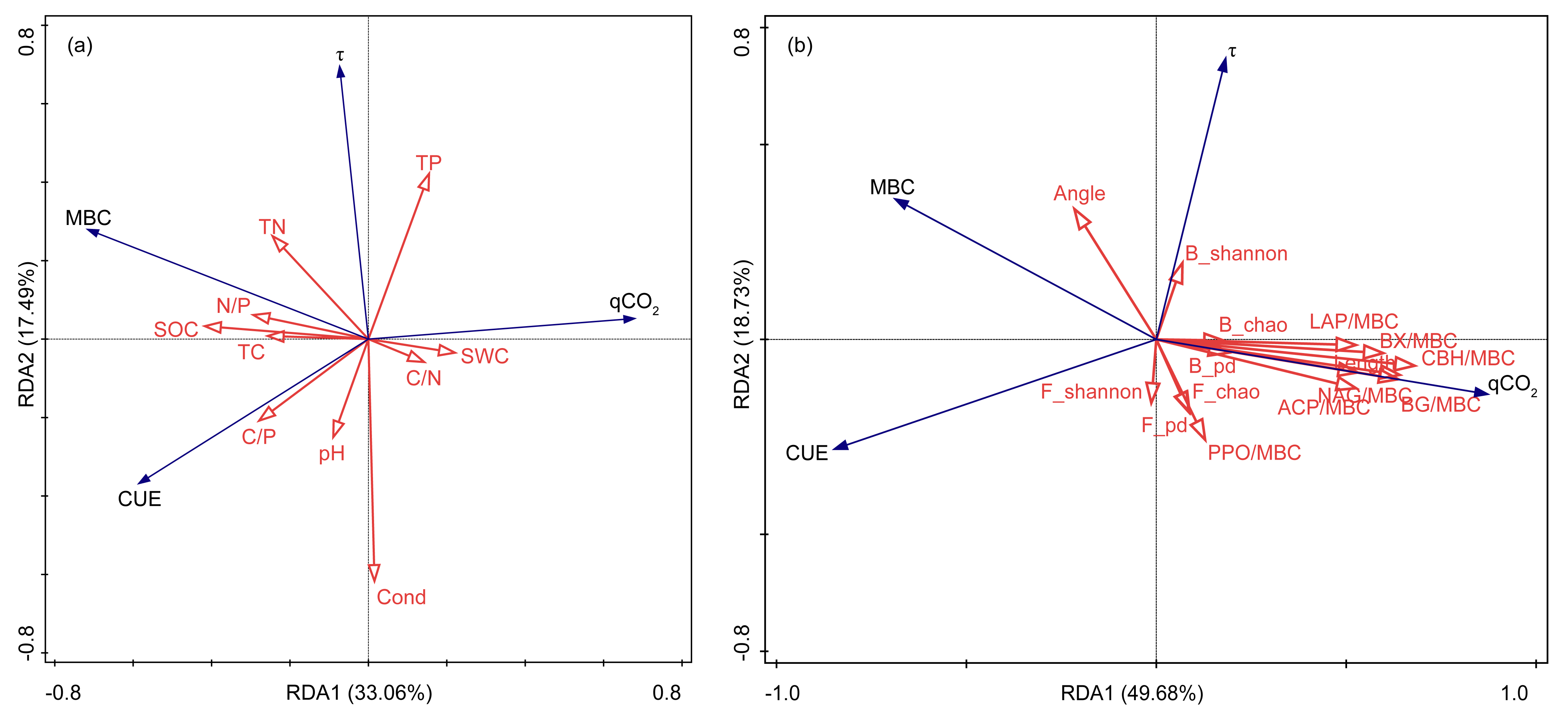

Supplement: Supplementary file 8 [file Image_8.JPEG]

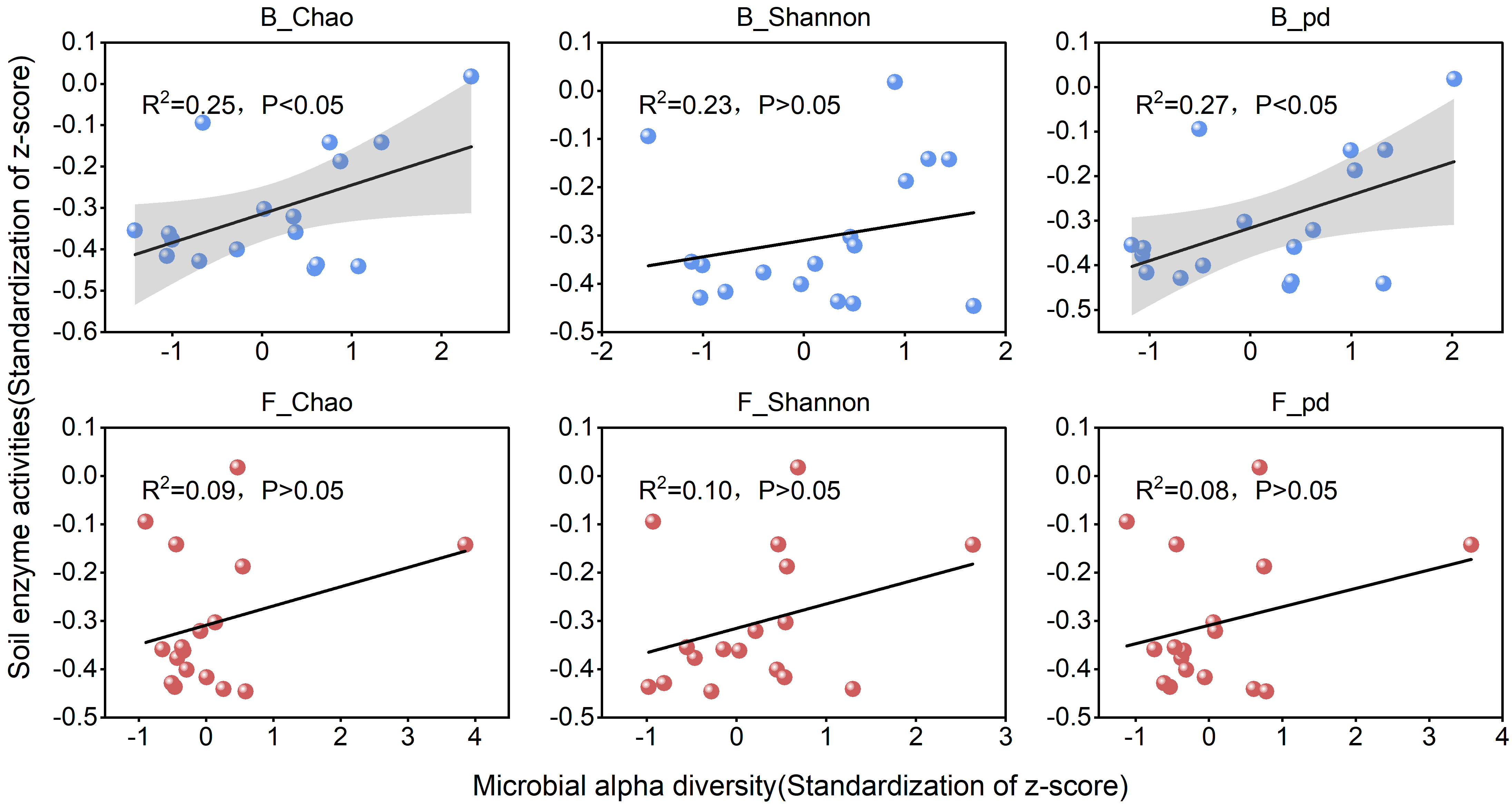

Supplement: Supplementary file 9 [file Image_9.JPEG]

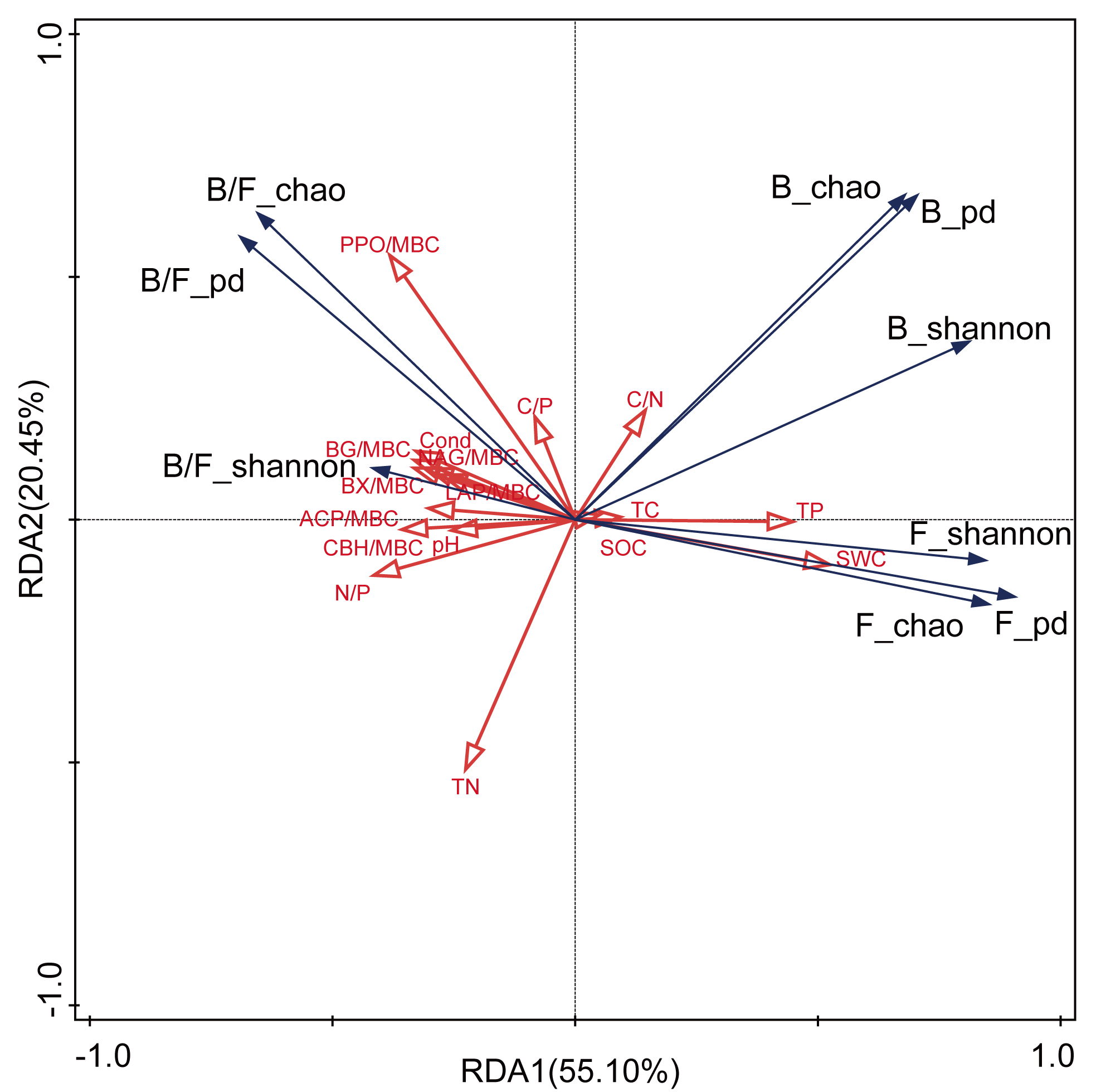

Supplement: Supplementary file 10 [file Image_10.JPEG]
