# Supplementary material for: Microbial community and soil enzyme activities driving microbial metabolic efficiency patterns in riparian soils of the Three Gorges Reservoir
Source: Front Microbiol. 2023 Apr 21;14:1108025. doi: 10.3389/fmicb.2023.1108025 (PMC10171112; doi:10.3389/fmicb.2023.1108025)
Supplement: Supplementary file 11 [file Data_Sheet_1.docx]

Supplementary Material

**Microbial community and soil enzyme activities driving microbial metabolic efficiency patterns in riparian soils of the Three Gorges Reservoir**

Yining Yang^1^, Yao Chen^1^, Zhe Li^2^, Yuanyuan Zhang^2^, Lunhui Lu^2,*^

^1^ Key Laboratory of Hydraulic and Waterway Engineering of the Ministry of Education, Chongqing Jiaotong University, Chongqing 400074, China

^2^ CAS Key Laboratory of Reservoir Water Environment, Chongqing Institute of Green and Intelligent Technology, Chinese Academy of Sciences, Chongqing 400714, China

^*^ Corresponding author: Lunhui Lu

E-mail address: [lulunhui@cigit.ac.cn](mailto:lulunhui@cigit.ac.cn)

1. **Supplementary Appendix S1**

**Detailed steps for the determination of soil TP by the SMT (Standards, Measurements and Testing) method developed under the framework of the European Standards and Testing Committee.**

Firstly, 200mg of the naturally dried soil sample was weighed in a porcelain crucible and cauterised in a muffle furnace at 450°C for 3h. After cooling, the sample was carefully poured into a 50mL centrifuge tube and 20mL of 3.5M HCl extract was added to the crucible in batches with a burette to transfer the sample and rinse until it was all transferred into the centrifuge tube, after which it was fixed flat in a constant temperature shaking incubator at 250r/min and 25°C for 16h. Immediately afterwards, the samples were centrifuged at 2000 g for 15 min and the total phosphorus in the supernatant was determined immediately by molybdenum antimony spectrophotometry method (Ruban et al., 2001; Sun et al., 2021).

1. **Supplementary Appendix S2**

**Detailed procedures for measuring and calculating the Microbial biomass carbon (MBC).**

The MBC was measured as follows:

It should be noted that two beakers need to be placed in the vacuum desiccator, one containing 25 mL of de-ethanolized chloroform (with a small amount of explosion-proof boiling particles) and the other containing 25 mL of soda lime solution. Before fumigation treatment of soil samples, a small amount of moistened filter paper is placed on the inner wall of the desiccator. The vacuum desiccator needs to be equipped with a built-in explosion-proof device.

Firstly, three portions of fresh samples equivalent to 10g of drying mass (passed through 2mm sieve) were weighed and put into different glass beakers, and then placed in a vacuum desiccator. Then, the desiccator was pumped into vacuum with a vacuum pump until the chloroform boiled and lasted for two minutes, the desiccator valve was closed, and the desiccator was placed in an incubation chamber at 25℃ ± 2 ℃ for 24 h under dark conditions. After the fumigation, remove the beaker with chloroform and the filter paper at the bottom from the desiccator, and then repeatedly evacuate (9 or 10 times, 2 min each time, it is better to open the lid of the desiccator completely after each evacuation) to remove the residual chloroform from the soil until the soil has no chloroform taste. The fumigated soil sample was transferred to a polyethylene plastic bottle (250 mL) without damage, 40 mL of potassium sulfate solution (c=0.5 mol/L) was added and shaken at 200 r/min for 60 min with a horizontal shaker, then the leachate was filtered with a 0.45um filter tip using a disposable plastic syringe. Three fresh unfumigated soil samples (10g dry basis weight) were weighed and placed in different polyethylene plastic bottles (250 mL) as control samples, and the unfumigated samples were extracted and filtered by the same method. The above extraction solution was aspirated to 2 mL and diluted a certain number of times to ensure that its organic carbon content was within the instrumental determination range. The organic carbon contents were analyzed with a Vario TOC Cube analyzer (Elemen-tar, Hanau, Germany).

The MBC is calculated as follows.

MBC = (Ec^CHCL3^-Ec^CK^)*N*f/0.45 (1)

Where Ec^CHCL3^ is the organic carbon content of leached fumigated soil samples (ug-C/g), Ec^CK^ is the organic carbon content of leached non-fumigated soil samples (ug-C/g), N is the dilution factor, and f is the water-to-soil ratio of fresh soil samples. 0.45 was calculated from the correlation between soil microbial biomass carbon (23 soils) obtained by fumigation culture method and fumigation leaching method.

**3 Supplementary Appendix S3**

**Bioinformatics analysis.**

The obtained sequences were quality-filtered and chimeras were identified with the QIIME2 pipeline (version 2) (Bolyen et al., 2019). All bioinformatics analyses were based on amplicon sequence variants (ASVs) (Callahan et al., 2017), using DATA2 denoising to remove any low-quality reads, and then clustering the eligible merged sequences into ASV (Callahan et al., 2016). Bacterial 16S rRNA gene sequences were matched in the Silva database (Release 132 http://www.arb-silva.de) and fungal ITS gene sequences were aligned with the Unite fungal database (Release 7.2 <http://unite.ut.ee/index.php>) (Quast et al., 2013). In this study, alpha diversity indices (Chao1, Shannon and phylogenetic diversity (PD)) were calculated based on 97% ASV similarity of the sequences. The *igraph* package in RStudio was used to calculate possible paired Spearman rank correlation matrices based on the ASV level. Only high Spearman correlation coefficients (|r|>0.8) and statistically significant (p<0.05) correlations were accepted for bacterial and fungal contribution network analysis to identify the main ecological clusters (modules or assemblies) of strongly correlated ASVs (Li et al., 2022). The co-occurrence network was visualized with Gephi software (version 0.9.5). The relative abundance of each module was calculated by averaging the standard relative abundances (z-score) of all taxa that belonged to each module (Delgado‐Baquerizo et al., 2018). We removed ASVs with relative abundance below 0.01% in bacterial and fungal communities (Ma et al., 2016) before performing a co-occurrence network analysis. Keystone taxa in microbial communities drive the composition and function of communities (Banerjee et al., 2018). Modules are highly connected regions of a network and are considered to be phylogenetically, evolutionarily, or functionally separate units (Olesen et al., 2007). Based on the node topology characteristics, we use intra-module connectivity (Zi) and inter-module connectivity (Pi) to infer the properties of the nodes in the network (Deng et al., 2012). The threshold values for Pi and Zi that we use here for network node classification are 0.62 and 2.5 respectively (Guimerà and Amaral, 2005; Guimerà et al., 2005). According to the simplified criteria, all species were sorted into four subcategories: peripherals, connectors, module hubs, and network hubs. Module hubs, connectors, and network hubs are usually classified as keystone taxa in microorganisms (Deng et al., 2012), as their disappearance may cause serious damage to the module or network.

| **Table S1. Geographic information of the sampling sites.** | | | | |
| --- | --- | --- | --- | --- |
| No | Sample sites | Latitude and longitude | Mainstream or Tributary | Segmentation |
| 1 | Zhutuo（ZT） | N29°1'00"E105°51'00" | Mainstream | Upstream |
| 2 | Mudong（MD） | N29°35'27"E106°50'16" | Mainstream |  |
| 3 | Fuling（FL） | N29°51'54"E107°32'23" | Mainstream | Midstream |
| 4 | Zhongxian（ZX） | N30°25'5.16"E108°11'54.24" | Mainstream |  |
| 5 | Wanzhou（WZ） | N30°54'57.24"E108°32'14.64" | Mainstream |  |
| 6 | Wenquan（WQ） | N31°20′1.3″E108°30′48.8″ | Tributary |  |
| 7 | Baijiaxi（BJX） | N31°11′7.6″E108°27′21.2″ | Tributary |  |
| 8 | Gaoyang（GY） | N31°5′48.2″E108°40′20.1″ | Tributary |  |
| 9 | Puan（PA） | N30°51'58.40"E108°54'42.16" | Tributary |  |
| 10 | Xinjing（XJ） | N30°55'14.39"E108°57'22.75" | Tributary |  |
| 11 | Fengjie（FJ） | N31°2'36.96"E109°34'58.44" | Mainstream | Downstream |
| 12 | Dachang（DC） | N31°13’26.3"E109°50'37.818" | Tributary |  |
| 13 | Shuanglong（SL） | N31°11′23.154″E109°52′31.296″ | Tributary |  |
| 14 | Shennongxi（SNX） | N31°03'42.22"E110°19'23.97" | Tributary |  |
| 15 | Badong（BD） | N31° 2'28.362"E110°19'26.640" | Mainstream |  |
| 16 | Yanduhe（YDH） | N31°09'28.14"E110°19'32.63" | Tributary |  |
| 17 | Guanzhuangping（GZP） | N31°0′29.454″E110°45′30.162″ | Tributary |  |
| 18 | Xiangxihekou（XXHK） | N30°57'34.200"E110°45'0.240" | Mainstream |  |
| 19 | Maoping（MP） | N30°51'17.130"E110°58'50.440" | Mainstream |  |
| 20 | Shandouping（SDP） | N30°49'52.780"E111° 2'59.800" | Mainstream |  |

| **Table S2. Results of principal components analysis (PCA) of soil physical and chemical properities, microbial alpha diversity** **(containing bacteria and fungi, respectively) and microbial metabolism efficiency across the 20 sampling sites.** | |
| --- | --- |
| **Factors** | **PC1** |
| **Soil physical and chemical properities** |  |
| pH | **-0.70***** |
| TC | **0.85***** |
| C/N | **0.70***** |
| C/P | **0.94***** |
| Cumulative (%) | 75.10 |
| **Microbial alpha diversity_Bacteria** |  |
| B_chao | **0.98***** |
| B_shannon | **0.90***** |
| B_pd | **0.96***** |
| Cumulative (%) | 91.34 |
| **Microbial alpha diversity_Fungi** |  |
| F_chao | **0.91***** |
| F_shannon | **0.93***** |
| F_pd | **0.94***** |
| Cumulative (%) | 89.75 |
| **Microbial metabolism efficiency** |  |
| CUE | **0.82***** |
| MBC | **0.88***** |
| qCO_2_ | **-0.91***** |
| Cumulative (%) | 70.64 |

Note: ***P < 0.001.

**Table S3. Multiple comparative analyses of microbial community composition in different zones of the TGR.**

| **Bacteria** | **Upstream** | **Midstream** | **Downstream** |
| --- | --- | --- | --- |
| Phylum | Proportion（Mean±SD, %） | | |
| p__Proteobacteria | 26.89±9.25 | 23.98±9.44 | 19.51±7.36 |
| p__Chloroflexi | 15.93±5.04 | 12.32±2.46 | 17.09±6.23 |
| p__Acidobacteriota | 14.54±3.64 | 14.49±5.75 | 13.69±4.45 |
| p__Actinobacteriota | 8.04±0.09**^b^** | 25.79±9.73**^a^** | 28.09±6.95**^a^** |
| p__Bacteroidota | 6.85±2.15**^a^** | 2.29±1.62**^b^** | 2.44±1.94**^b^** |
| p__Desulfobacterota | 6.65±2.56**^a^** | 1.87±1.75**^b^** | 0.87±0.76**^b^** |
| p__Nitrospirota | 4.09±0.16**^a^** | 1.49±0.48**^b^** | 0.87±0.52**^c^** |
| p__MBNT15 | 1.96±0.09**^a^** | 0.12±0.16**^b^** | 0.21±0.28**^b^** |
| p__Firmicutes | 1.89±0.45 | 3.72±1.53 | 3.05±1.87 |
| p__Spirochaetota | 1.86±0.17**^a^** | 0.04±0.10**^b^** | 0.00±0.01**^b^** |
| p__Nitrospinota | 1.51±0.38**^a^** | 0.06±0.08**^b^** | 0.02±0.06**^b^** |
| p__Myxococcota | 1.46±0.23 | 3.28±2.26 | 2.40±0.56 |
| p__Gemmatimonadota | 1.24±0.20 | 2.54±1.37 | 2.25±1.18 |
| p__Latescibacterota | 1.19±0.65 | 0.88±0.68 | 0.86±0.60 |
| p__Methylomirabilota | 1.03±0.32 | 2.77±1.49 | 3.50±2.33 |
| p__GAL15 | 0.03±0.04 | 0.09±0.16 | 1.25±2.24 |
| others | 4.84±0.24 | 4.27±1.61 | 3.90±1.35 |
| **Fungi** | **Upstream** | **Midstream** | **Downstream** |
| Phylum | Proportion（Mean±SD, %） | | |
| p__unclassified_k__Fungi | 34.40±26.16 | 31.60±16.33 | 14.14±12.66 |
| p__Ascomycota | 32.64±12.24 | 33.69±20.96 | 40.99±34.71 |
| p__Rozellomycota | 23.80±9.29**^a^** | 5.20±4.86**^b^** | 0.33±0.30**^c^** |
| p__Basidiomycota | 5.76±2.63 | 17.26±14.95 | 21.74±27.31 |
| p__Mortierellomycota | 2.03±2.67 | 10.53±10.15 | 20.19±25.53 |
| p__Chytridiomycota | 1.14±0.78**^a^** | 0.47±0.47**^ab^** | 0.16±0.14**^b^** |
| p__Calcarisporiellomycota | 0.06±0.08 | 0.64±1.14 | 1.66±3.83 |
| others | 0.17±0.03 | 0.61±0.43 | 0.79±0.58 |

Note: **^abc^**The different letters represent significant differences (p < 0.05) between upstream, midstream and downstream. SD: standard deviation.

| **Table S4. Correlation coefficients (Pearson’s rank correlation coefficients) between the relative abundance of key microorganisms (containing bacteria and fungi, genus level) and microbial metabolism efficiency as well as soil microbial trait-based strategies.** | | | | | | | |
| --- | --- | --- | --- | --- | --- | --- | --- |
|  | Key Genus | CUE | MBC | τ | qCO_2_ | Length | Angle |
| Bacteria | Vicinamibacterales | -0.29 | -0.23 | 0.09 | 0.25 | **0.57**** | 0.30 |
|  | Gaiellales | 0.17 | -0.03 | -0.04 | -0.06 | **-0.62**** | -0.41 |
|  | Bradyrhizobium | 0.11 | 0.44 | -0.07 | -0.12 | -0.03 | 0.02 |
|  | Ellin6067 | 0.01 | 0.42 | -0.02 | -0.11 | 0.03 | -0.04 |
|  | Rhizobiaceae | 0.10 | 0.22 | 0.21 | -0.18 | 0.20 | -0.21 |
|  | Bacillus | 0.10 | -0.21 | -0.14 | -0.08 | 0.04 | 0.33 |
|  | Delftia | -0.11 | -0.32 | 0.28 | 0.16 | -0.01 | -0.34 |
| Fungi | unclassified_k__Fungi | 0.23 | **0.53*** | 0.01 | -0.14 | -0.02 | 0.29 |
|  | Basidiomycota | -0.31 | -0.10 | 0.05 | 0.30 | -0.03 | -0.19 |
|  | Cadophora | 0.06 | -0.12 | -0.16 | -0.01 | **0.56**** | 0.15 |
|  | Talaromyces | -0.20 | -0.31 | -0.14 | 0.07 | 0.13 | 0.32 |
|  | Mortierella | 0.20 | -0.09 | 0.23 | -0.31 | -0.29 | -0.38 |
|  | Lasiosphaeriaceae | 0.24 | -0.09 | -0.28 | 0.06 | -0.11 | 0.04 |
|  | Staphylotrichum | -0.01 | -0.06 | -0.30 | 0.29 | -0.08 | -0.14 |
|  | Paraconiothyrium | -0.36 | 0.04 | 0.08 | 0.26 | -0.10 | 0.09 |

Note: *P < 0.05, **P < 0.01.

| **Table S5. Correlation coefficients (Pearson’s rank correlation coefficients) between the relative abundance of dominant microorganisms (containing bacteria and fungi, phylum level) and microbial metabolism efficiency as well as soil microbial trait-based strategies.** | | | | | | | |
| --- | --- | --- | --- | --- | --- | --- | --- |
|  | Dominant Phyla | CUE | MBC | τ(h-1) | qCO_2_ | Length | Angle |
| Bacteria | p__Actinobacteriota | 0.28 | -0.06 | -0.03 | -0.27 | **-0.65**** | -0.30 |
|  | p__Proteobacteria | 0.07 | 0.28 | -0.06 | -0.28 | 0.14 | 0.30 |
|  | p__Chloroflexi | -0.23 | -0.09 | 0.38 | 0.31 | 0.03 | -0.20 |
|  | p__Acidobacteriota | -0.11 | -0.07 | -0.06 | 0.22 | 0.22 | 0.30 |
|  | p__Firmicutes | 0.18 | -0.16 | -0.16 | -0.15 | -0.03 | 0.18 |
|  | p__Methylomirabilota | -0.08 | -0.21 | 0.00 | 0.08 | 0.01 | -0.15 |
|  | p__Bacteroidota | -0.21 | -0.03 | -0.11 | 0.09 | 0.28 | -0.01 |
|  | p__Myxococcota | 0.17 | **0.52*** | -0.12 | -0.24 | -0.09 | 0.23 |
|  | p__Gemmatimonadota | 0.14 | -0.26 | 0.08 | -0.19 | -0.08 | 0.07 |
|  | p__Desulfobacterota | -0.28 | 0.07 | -0.10 | **0.45*** | **0.51*** | 0.07 |
|  | p__Nitrospirota | -0.35 | -0.08 | -0.07 | **0.48*** | **0.63**** | 0.13 |
| Fungi | p__Ascomycota | 0.11 | -0.15 | -0.09 | -0.22 | 0.15 | 0.41 |
|  | p__unclassified_k__Fungi | -0.01 | **0.47*** | 0.02 | 0.14 | 0.04 | 0.03 |
|  | p__Basidiomycota | -0.26 | -0.10 | 0.03 | 0.29 | -0.08 | -0.08 |
|  | p__Mortierellomycota | 0.26 | -0.05 | -0.02 | -0.22 | -0.33 | -0.41 |
|  | p__Rozellomycota | -0.22 | -0.01 | -0.21 | 0.30 | **0.45*** | 0.02 |
|  | p__Calcarisporiellomycota | -0.28 | -0.27 | 0.07 | **0.49*** | -0.10 | **-0.60**** |

Note: *P < 0.05, **P < 0.01.

| **Table S6. Proportion of dominant bacteria and fungi (phylum level).** | | |
| --- | --- | --- |
|  | Dominant Phyla | Proportion（%） |
| Bacteria | p__Actinobacteriota | 25.41 |
|  | p__Proteobacteria | 21.65 |
|  | p__Chloroflexi | 15.35 |
|  | p__Acidobacteriota | 14.07 |
|  | p__Firmicutes | 3.30 |
|  | p__Methylomirabilota | 3.00 |
|  | p__Bacteroidota | 2.69 |
|  | p__Myxococcota | 2.63 |
|  | p__Gemmatimonadota | 2.33 |
|  | p__Desulfobacterota | 1.74 |
|  | p__Nitrospirota | 1.40 |
|  | others | 6.42 |
| Fungi | p__Ascomycota | 36.64 |
|  | p__unclassified_k__Fungi | 23.83 |
|  | p__Basidiomycota | 17.74 |
|  | p__Mortierellomycota | 14.76 |
|  | p__Rozellomycota | 4.89 |
|  | p__Calcarisporiellomycota | 1.12 |
|  | others | 1.02 |
| Note: Classify less than 1% as others. | | |

| **Table S7. Fit index value of structural equation model.** | | |
| --- | --- | --- |
| **（a）bacteria** |  |  |
| Fitment indicators | Recommended values | Fitted values |
| χ^2^ | The smaller the better | 0.063 |
| χ^2^/df | <3.0 | 0.063 |
| p | >0.05 | 0.802 |
| GFI | >0.9 | 0.998 |
| AGFI | >0.8 | 0.980 |
| CFI | >0.9 | 1.000 |
| RMSEA | <0.08 | 0.000 |
| AIC | The smaller the better | 18.063 |
| BIC | The smaller the better | 25.562 |
| **（b）fungi** |  |  |
| Fitment indicators | Recommended values | Fitted values |
| χ^2^ | The smaller the better | 0.092 |
| χ^2^/df | <3.0 | 0.092 |
| p | >0.05 | 0.762 |
| GFI | >0.9 | 0.997 |
| AGFI | >0.8 | 0.971 |
| CFI | >0.9 | 1.000 |
| RMSEA | <0.08 | 0.000 |
| AIC | The smaller the better | 18.092 |
| BIC | The smaller the better | 25.591 |


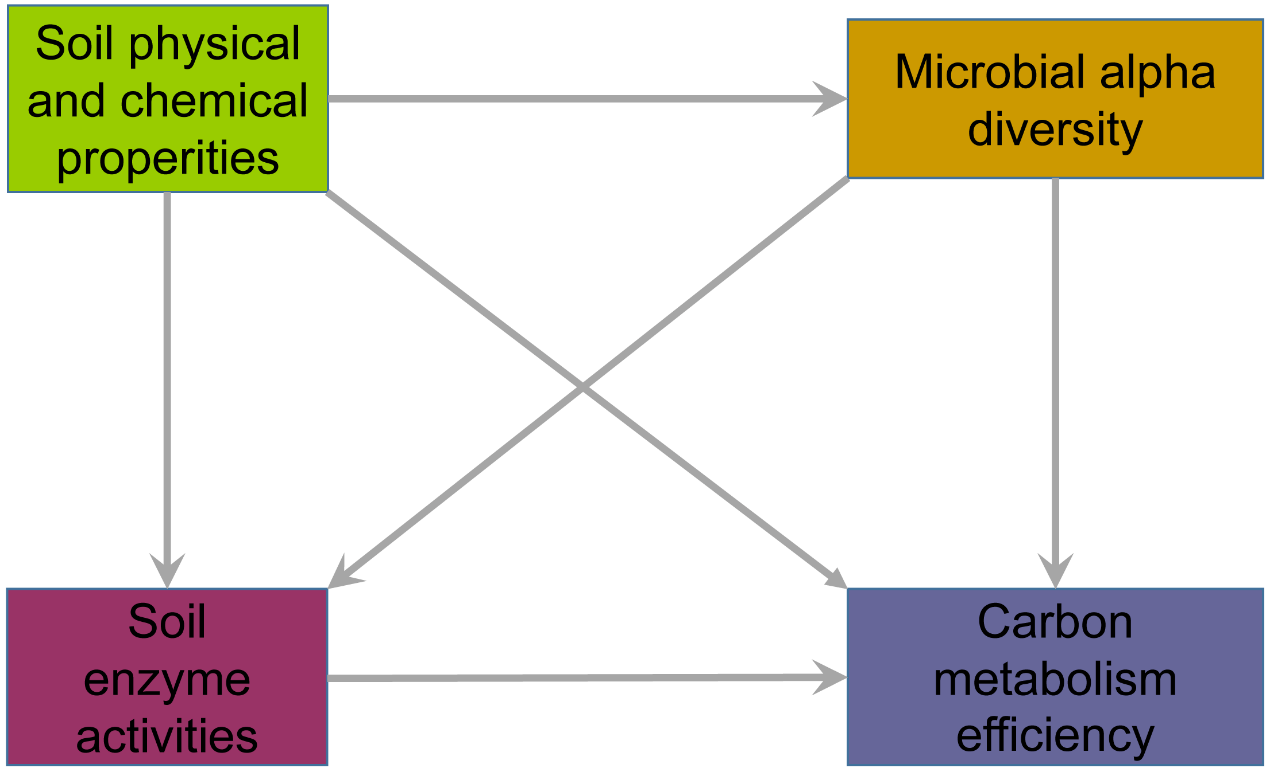


**Supplementary Figure S1.** **Hypothesized path model structure to evaluate abiotic and biotic factors direct and indirect effects of on microbial metabolic efficiency.** **We infer that: 1) Soil physical and chemical properties can directly affect microbial α diversity, soil enzyme activities and microbial metabolic efficiency; 2) Microbial α diversity drives soil enzyme activities and microbial metabolic efficiency, and the influence of bacterial and fungal α diversity on them is different; 3) Soil enzyme activities have direct effect on microbial metabolic efficiency.**


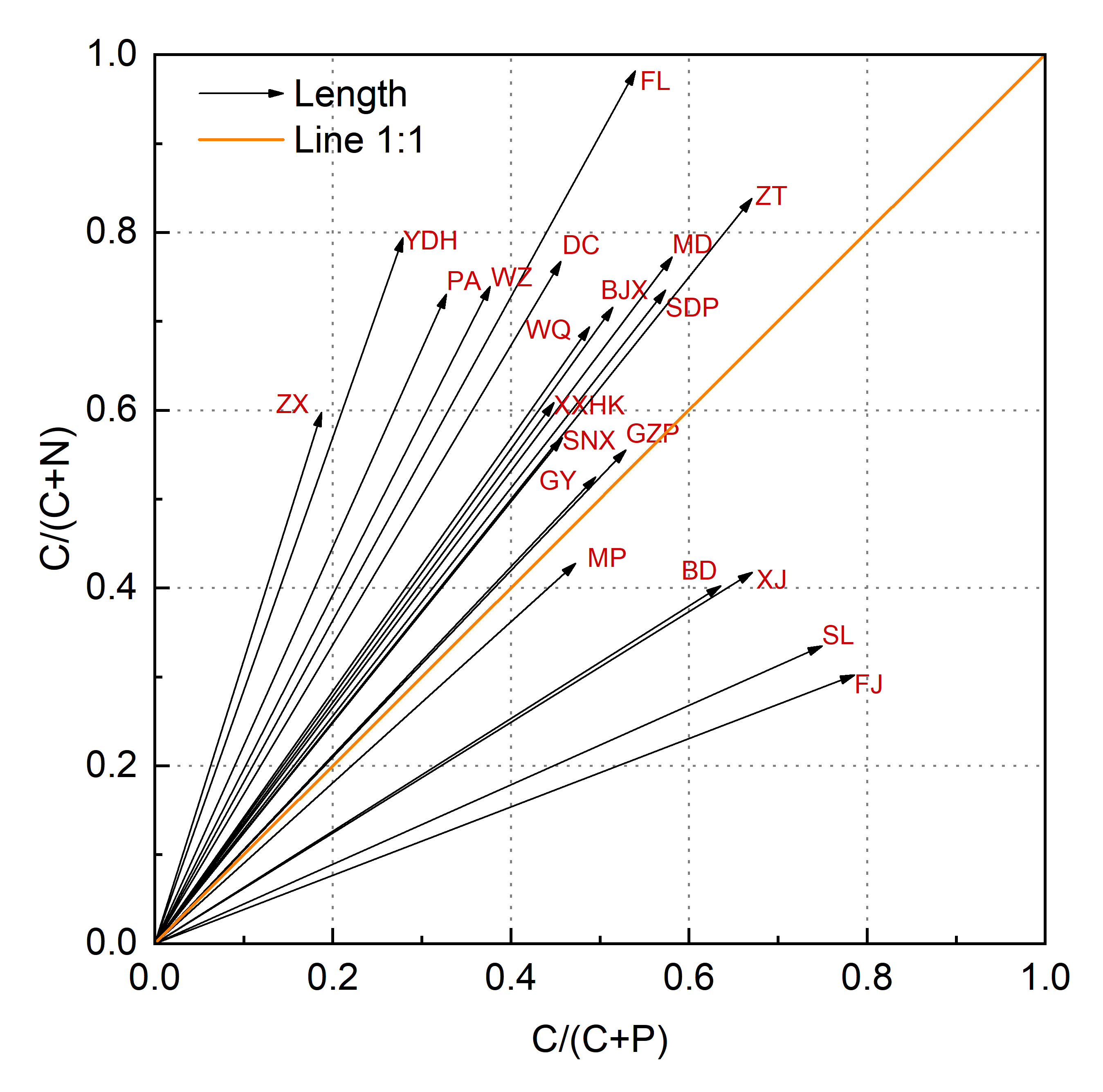


**Supplementary Figure S2. Extracellular C, N, and P acquire enzymatic stoichiometry relationships.**


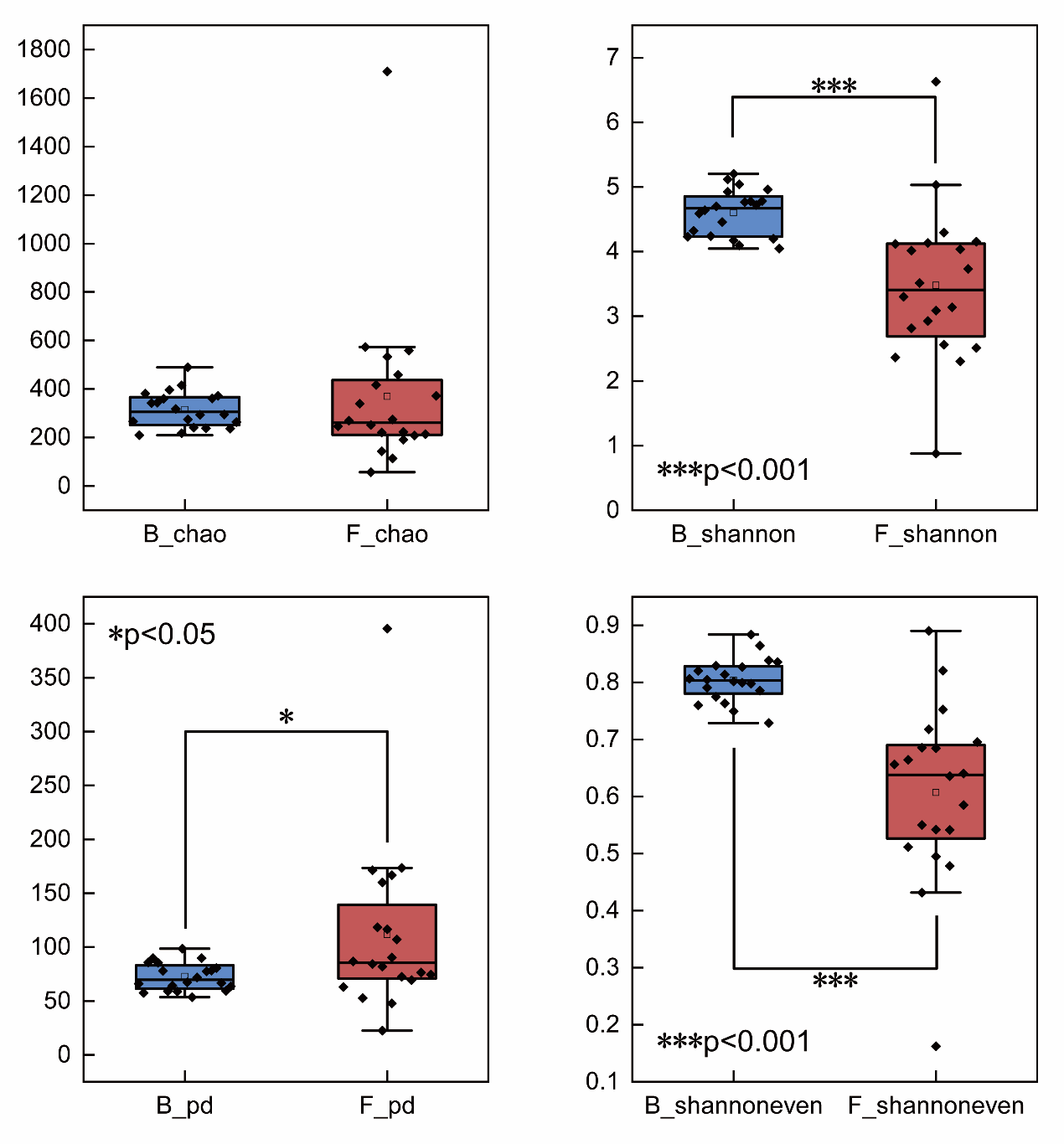
**Supplementary Figure S3. Overall distribution of bacterial and fungal alpha diversity indicators (including Chao, Shannon, pd and Shannoneven indices) and comparative analysis between them. *P < 0.05, **P < 0.01, ***P < 0.001.**


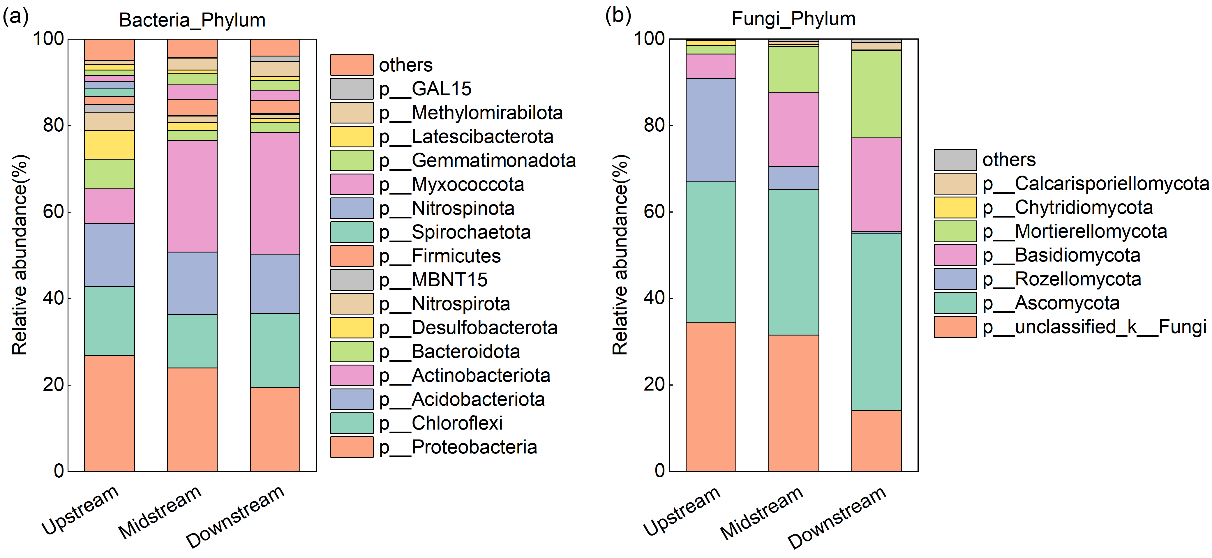


**Supplementary Figure S4. Proportion of microorganisms (including bacteria and fungi, phylum level) in riparian zones along the Three Gorges Reservoir. (a) Percentage of bacteria at the phylum level (upstream, midstream and downstream). (b) The representation of fungi at the phylum level (upstream, midstream and downstream). Phyla with relative abundance less than 0.01% at the microbial community phylum level are classified as others in the Figure.**


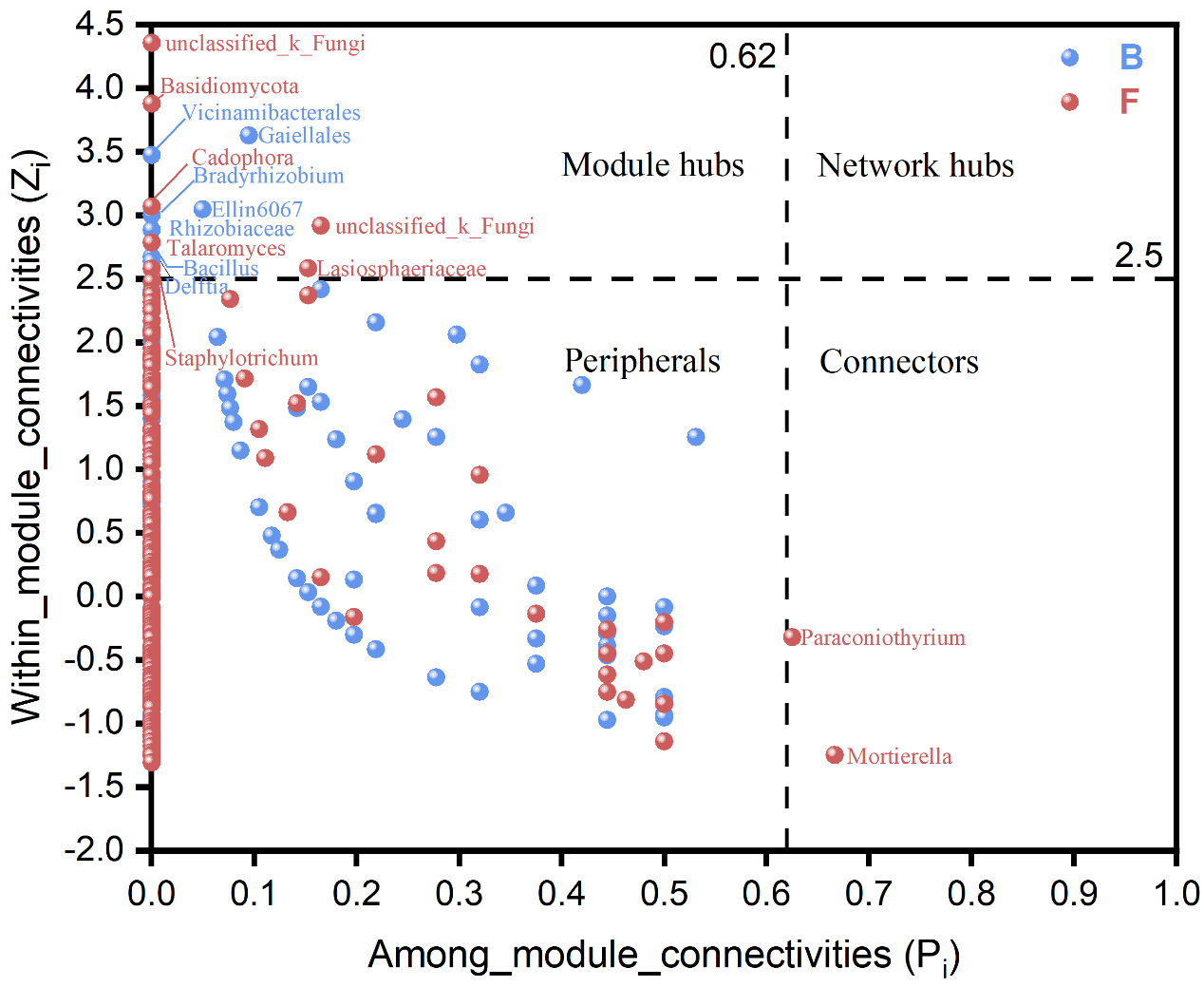


**Supplementary Figure S5. Z_i_-P_i_ plot showing the distribution of ASVs based on their topological roles in networks between bacteria and fungi. Each symbol represented an ASV in the bacterial (blue circle) or fungal (red circle) network. The threshold values of Z_i_ and P_i_ for categorizing ASVs were 2.5 and 0.62, respectively.**


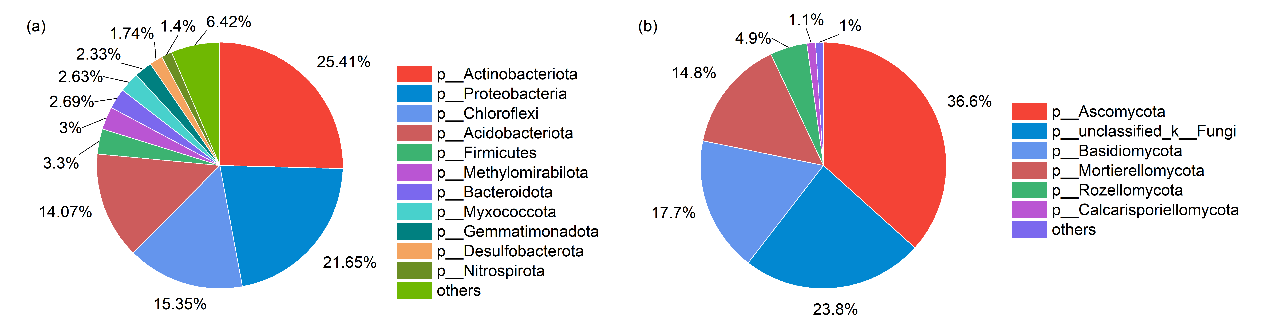


**Supplementary Figure S6. Percentage of dominant phyla level in the Three Gorges Reservoir Area riparian zone. (a) The proportion of dominant phyla in bacterial communities. (b) The proportion of dominant phyla in fungal communities.**


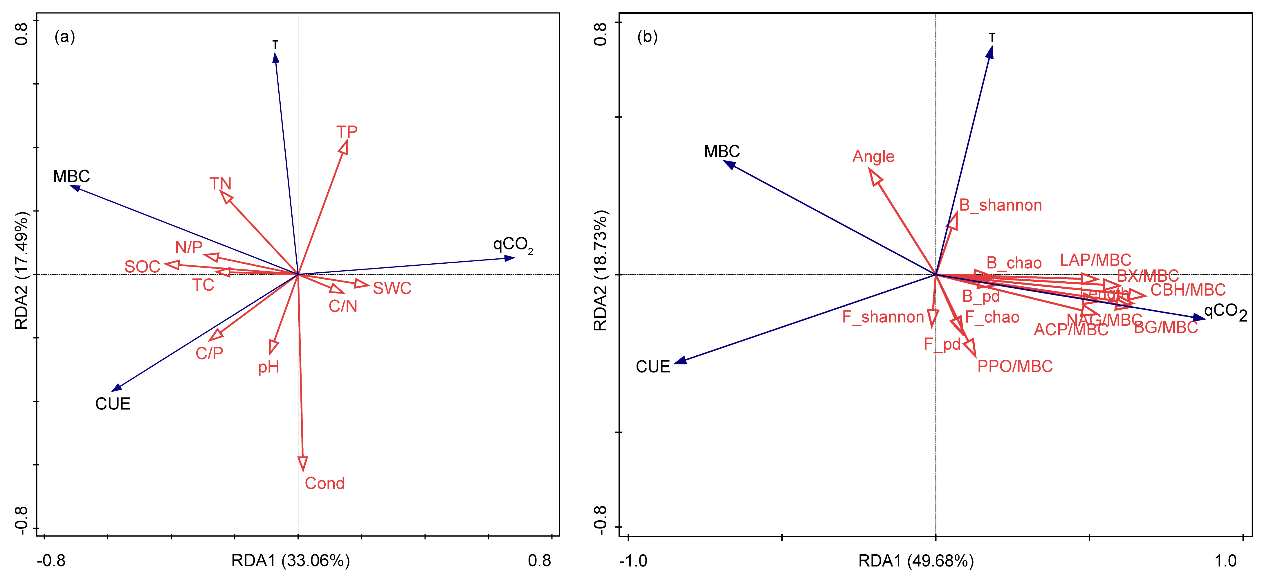


**Supplementary Figure S7. Redundancy Analysis (RDA). (a) Redundancy analysis between microbial metabolism efficiency and abiotic factors. (b) Redundancy analysis between microbial metabolism efficiency and biotic factors.**


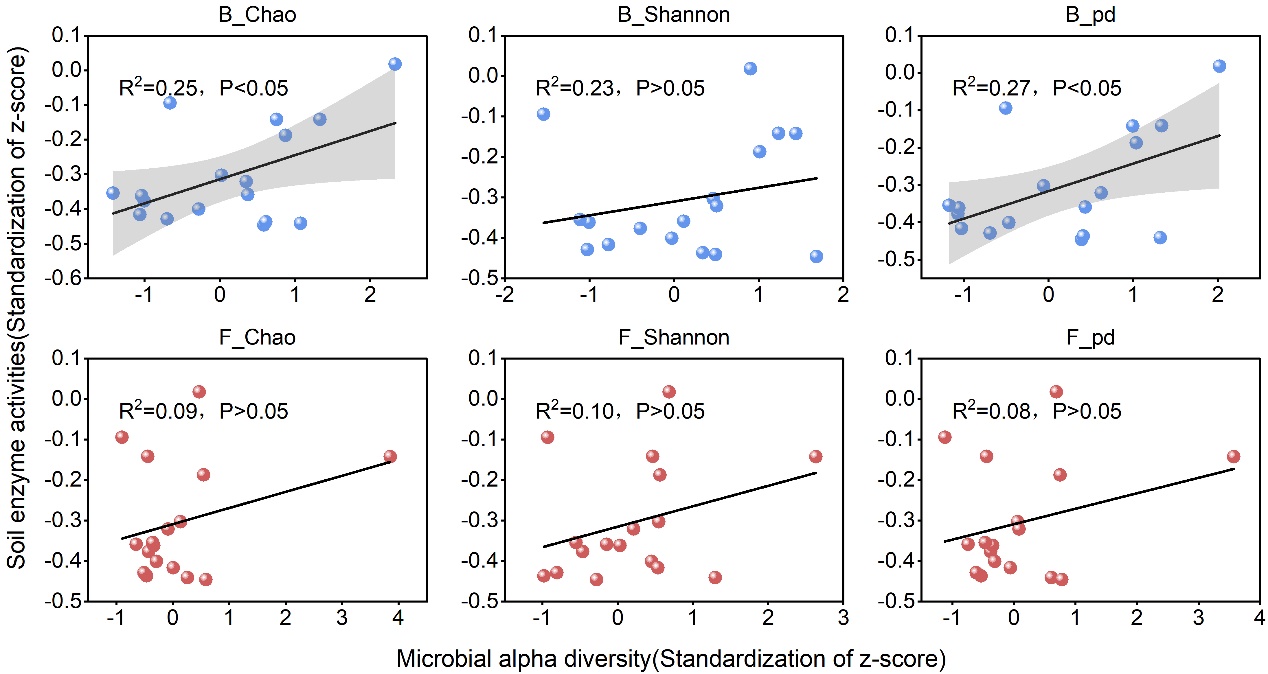


**Supplementary Figure S8. Linear fit of microbial alpha diversity to soil enzyme activities. Shaded areas show 95% confidence intervals of the fit.**


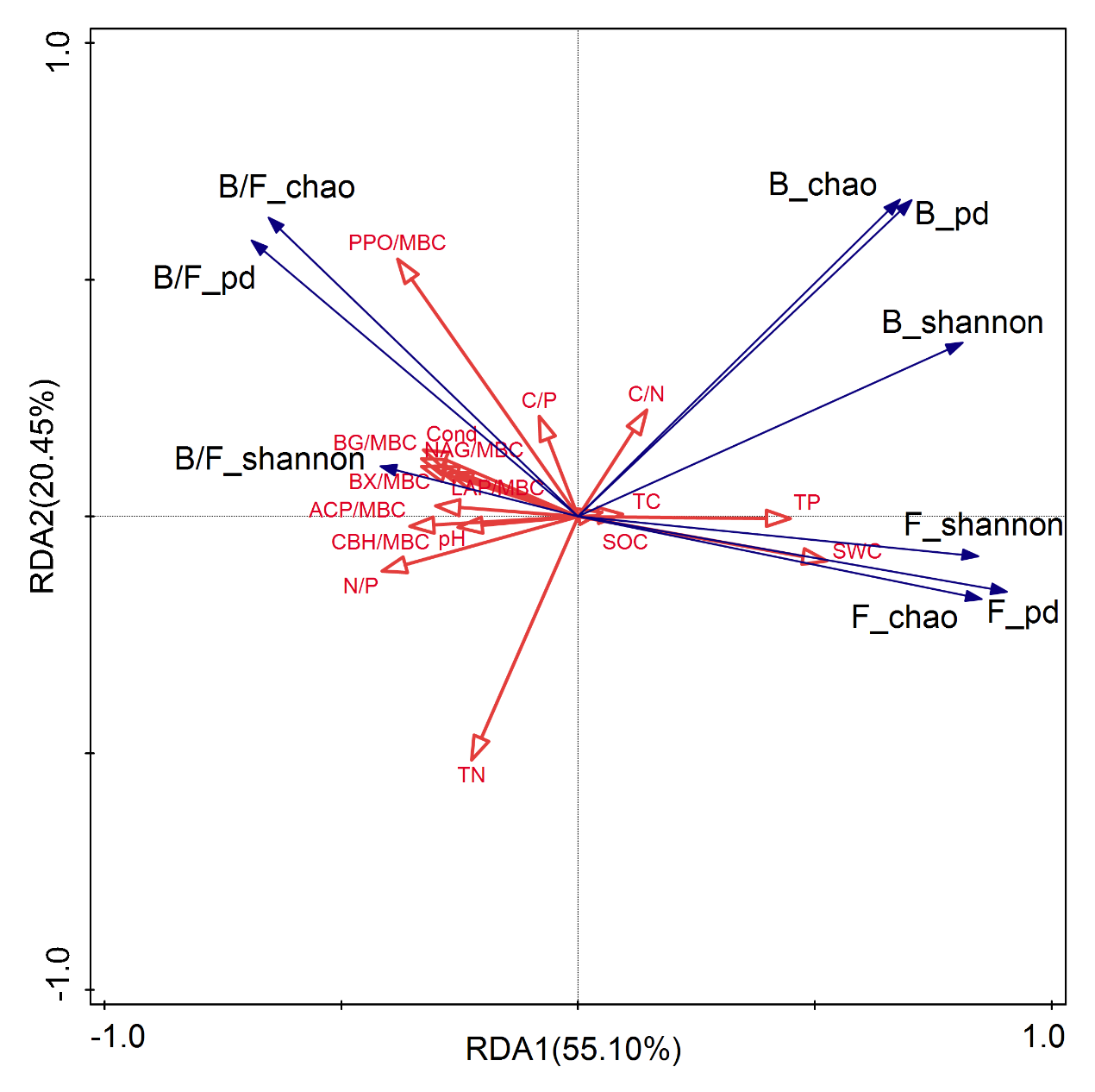


**Supplementary Figure S9. Redundancy analysis between microbial alpha diversity and soil enzyme activity, soil physical and chemical properties.**

**References**

Banerjee, S., Schlaeppi, K., and van der Heijden, M.G.A. (2018). Keystone taxa as drivers of microbiome structure and functioning. *Nat. Rev. Microbiol.* 16, 567-576.

Bolyen, E., Rideout, J.R., Dillon, M.R., Bokulich, N.A., Abnet, C.C., Al-Ghalith, G.A., et al. (2019). Reproducible, interactive, scalable and extensible microbiome data science using QIIME 2. *Nat. Biotechnol.*, 1-5.

Callahan, B.J., McMurdie, P.J., and Holmes, S.P. (2017). Exact sequence variants should replace operational taxonomic units in marker-gene data analysis. *ISME J.* 11, 2639-2643.

Callahan, B.J., McMurdie, P.J., Rosen, M.J., Han, A.W., Johnson, A.J.A., and Holmes, S.P. (2016). DADA2: High resolution sample inference from Illumina amplicon data. *Nat. Methods* 13, 581-583.

Delgado‐Baquerizo, M., Reith, F., Dennis, P.G., Hamonts, K., Powell, J.R., Young, A., et al. (2018). Ecological drivers of soil microbial diversity and soil biological networks in the Southern Hemisphere. *Ecology* 99 3, 583-596.

Deng, Y., Jiang, Y. H., Yang, Y., He, Z., Luo, F., and Zhou, J. (2012). Molecular ecological network analyses. *BMC Bioinformatics* 13, 113 (2012).

Guimerà, R., and Amaral, L.A.N. (2005). Cartography of complex networks: modules and universal roles. *J. Stat. Mech: Theory Exp.* 2005, P02001.

Guimerà, R., Mossa, S., Turtschi, A., and Amaral, L.A.N. (2005). The worldwide air transportation network: Anomalous centrality, community structure, and cities' global roles. *Proc. Natl. Acad. Sci. U. S. A.* 102, 7794-7799.

Li, H., Li, Z., Tang, Q., Li, R., and Lu, L. (2022). Local-Scale Damming Impact on the Planktonic Bacterial and Eukaryotic Assemblages in the upper Yangtze River. *Microb. Ecol.* doi: 10.1007/s00248-022-02012-w.

Ma, B., Wang, H. Z., Dsouza, M., Lou, J., He, Y., Dai, Z., et al. (2016). Geographic patterns of co-occurrence network topological features for soil microbiota at continental scale in eastern China. *ISME J.* 10, 1891-1901.

Olesen, J.M., Bascompte, J., Dupont, Y.L., and Jordano, P. (2007). The modularity of pollination networks. *PNAS.* 104, 19891-19896.

Quast, C., Pruesse, E., Yilmaz, P., Gerken, J., Schweer, T., Yarza, P., et al. (2013). The SILVA ribosomal RNA gene database project: improved data processing and web-based tools. *Nucleic Acids Res.* 41, D590 -D596.

Ruban, V., López-Sánchez, J.F., Pardo, P., Rauret, G., Muntau, H., and Quevauviller, P. (2001). Harmonized protocol and certified reference material for the determination of extractable contents of phosphorus in freshwater sediments – A synthesis of recent works. *Fresenius' J. Anal. Chem.* 370, 224-228.

Sun, W., Yang, K., Li, R., Chen, T., Xia, L., Wang, Z., and Sun, X. (2021). The spatial distribution characteristics of typical pathogens and nitrogen and phosphorus in the sediments of Shahe Reservoir and their relationships. *Sci. Rep.* 11. doi:10.1038/s41598-021-01252-z
